# Subduction legacies in the mantle transition zone modulate intraplate oceanic volcanism

**DOI:** 10.1038/s41467-026-73403-7

**Published:** 2026-05-18

**Authors:** Jianfeng Yang, Manuele Faccenda, Christine M. Meyzen, Andrea Marzoli, Liang Zhao

**Affiliations:** 1 State Key Laboratory of Lithospheric and Environmental Coevolution, Institute of Geology and Geophysics, Chinese Academy of Sciences, Beijing, China; 2 College of Earth and Planetary Sciences, University of Chinese Academy of Sciences, Beijing, China; 3 Dipartimento di Geoscienze, Università di Padova, Padova, Italy; 4 Dipartimento Territorio e Sistemi Agro-Forestali, Università di Padova, Padova, Italy; 5 Key Laboratory of Deep Petroleum Intelligent Exploration and Development, Institute of Geology and Geophysics, Chinese Academy of Sciences, Beijing, China

**Keywords:** Geodynamics, Geochemistry

## Abstract

How oceanic crust forms and intraplate volcanism arises remains central to resolving the mechanisms driving Earth’s dynamic evolution. Anomalously thick oceanic crust is conventionally attributed to thermal mantle plumes, yet large igneous provinces such as the Azores Plateau, with its 8–30 km thick crust, dispersed volcanism, and distinctive water-rich geochemical signatures, challenge this paradigm. Here we use geodynamic numerical models to show that a migrating ridge over a locally hydrated layer (0.1–0.4 wt.% H₂O), generated by dehydration of the Mantle Transition Zone (MTZ), can trigger upwelling and melting sufficient to produce a 10–20 km-thick crust. This mechanism accounts for the plateau’s anomalous crustal thickness, long-lived volcanism, and volatile-rich mantle source. We propose that recycled water, a subduction legacy stored in the MTZ, acts as a primary driver of intraplate volcanism, providing an alternative to the classical stationary mantle plume model. This mechanism may also help explain the widespread contamination of large-scale upper mantle domains by subduction-related fluid signatures, as in the DUPAL and South Atlantic domains.

## Abstract (web summary)

Subduction-recycled water trapped in the mantle transition zone can be drawn up by a migrating mid-ocean ridge, triggering deep melting that forms thick oceanic plateau.

## Introduction

On Earth, the largest volumes of volcanism (~10^6^–10^7^ km^3^) are generated during geologically brief, cataclysmic events that build oceanic plateaus and continental flood basalts. These outbursts are widely attributed to the arrival of a mantle plume head, an anomalously hot, buoyant upwelling from the deep mantle that triggers massive melting upon impinging on the lithosphere^1^. Oceanic plateaus are often regarded as the most unequivocal surface manifestation of mantle plumes, i.e., thermally buoyant upwellings originating from a thermal boundary layer, most commonly the core-mantle boundary (CMB), characterized by potential temperature anomalies of 200–300 °C above ambient mantle. However, despite decades of investigation, multidisciplinary evidence has yet to conclusively support the plume head hypothesis for any known oceanic plateau^2,3^.

**Fig. 1:**
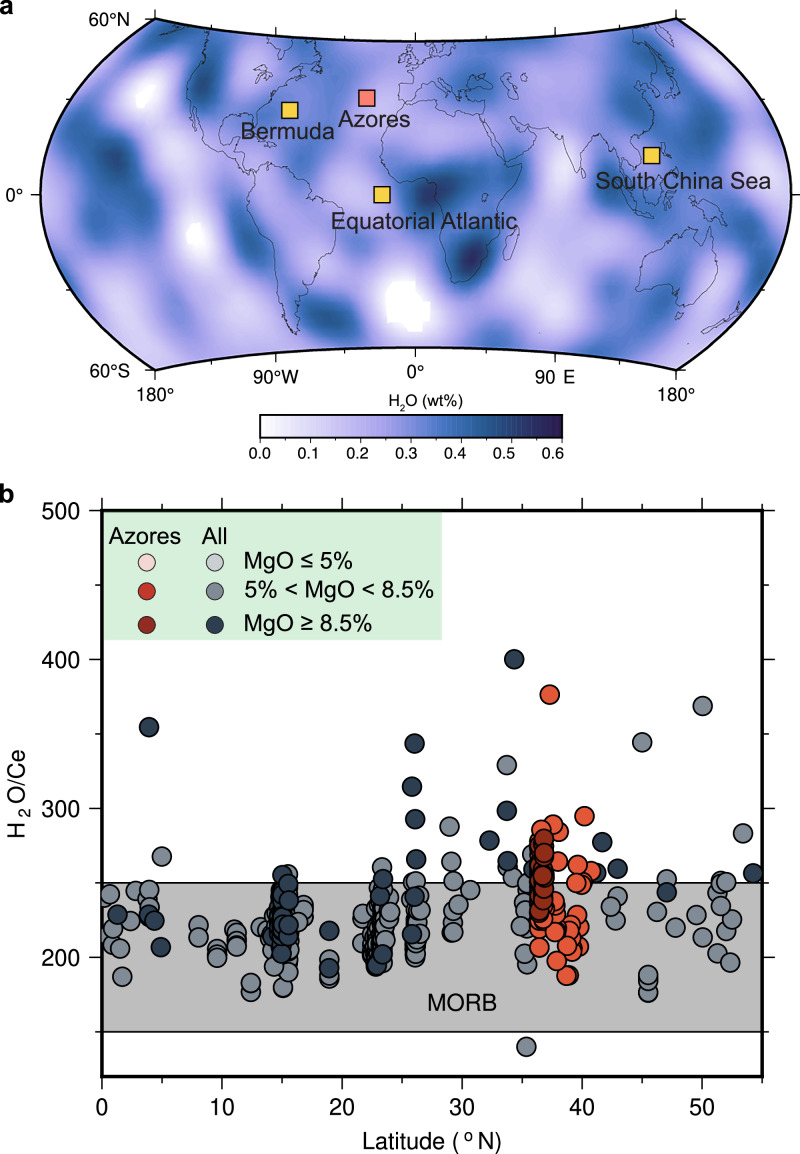
Water in the mantle transition zone and in MORBs from the Mid-Atlantic Ridge. **a** Seismic-experimentally constrained water contents at the upper MTZ^67^. The yellow squares indicate previously identified water-rich basalts in Bermuda^45^, Equatorial Atlantic^52^, and South China Sea^47^, where water is probably originating from the MTZ; basalts at Azores (pink square) are also water-rich^6,12^. **b** Latitudinal profile of H₂O/Ce ratios in MORB along the Mid-Atlantic Ridge, with MgO content indicated by the colour scale^54^. The grey shading zone indicates a background H₂O/Ce ratio (150–250). MORB, mid-ocean ridge basalt; MTZ, mantle transition zone.

The Azores Plateau, a now-rifted region of 8–30 km thick oceanic crust located at the junction of the North American, Eurasian, and Nubian plates, offers a rare natural laboratory for testing plume-driven versus volatile-enriched, fertile-mantle models^4–6^ (Fig. 1). Its unusually thick crust is often attributed to a hot plume, but the persistence of volcanism since about 39 Ma^7^, combined with overlapping eruption ages across the archipelago, and the lack of a clear, long-term age-progressive hotspot track^5,8^, challenges key predictions of the classical stationary plume model. This complexity is compounded by the strong tectonic control on recent volcanism, which occurs predominantly along structures such as the Terceira Rift^9,10^. Seismic imaging reveals only shallow upper mantle low-velocity anomalies, with its connection to the lower mantle remaining inconclusive^11^ (Supplementary Fig. 1). Sr-Nd-Pb-Hf isotopic systematics instead indicate contributions from recycled, volatile-rich subducted oceanic material in the mantle source^6,12–14^, consistent with elevated H₂O/Ce ratios of both ocean-island basalts (OIBs) and mid-ocean ridge basalts (MORBs) from the Azores compared to background MORB ratios to the south^6,12^ (Fig. 1). In addition, mantle temperatures inferred for Azores lavas assuming an anhydrous source lie at the lower bound of the global OIB range, overlapping substantially with the ambient mantle (1342–1461 °C)^15^.

These features point toward an origin involving a hydrated, fertile mantle source rather than a purely thermal anomaly. A compelling reservoir for such water is the mantle transition zone (MTZ, at depths of 410–660 km), where nominally anhydrous minerals (NAMs) can store up to 1–3 wt.% H₂O, far exceeding those of the upper and lower mantle (typically ≤0.1 wt.% H₂O)^16^. When water-rich MTZ material crosses the 410-km discontinuity and transforms to olivine, the excess water is expelled. At ambient mantle temperatures (~1800 K) exceeding the wet solidus (~1500–1600 K)^17,18^, this triggers dehydration melting, which produces a small fraction of hydrous melt that accumulates atop the 410-km discontinuity. Seismic imaging consistently revealed a global low-velocity layer ~20–100 km thick atop the 410-km discontinuity, interpreted as a hydrous partially molten layer^19–21^ (Fig. 1). Such a layer, being more buoyant than its dry counterpart^22,23^, could promote mantle upwelling and, in turn, trigger intraplate volcanism^24^.

To explore alternatives to plume-driven formation of anomalously thick crust, we develop geodynamic models that couple hydrogen partitioning in NAMs with hydrous melting and melt extraction. Our results indicate that a thin, partially molten layer atop the MTZ can sustain excess crustal production in intraplate, ridge-proximal settings. This mechanism reproduces the observed plateau thickness, providing a viable alternative, non-plume explanation for its origin.

## Results and Discussion

### Wet melting generates anomalously thick crust

**Fig. 2:**
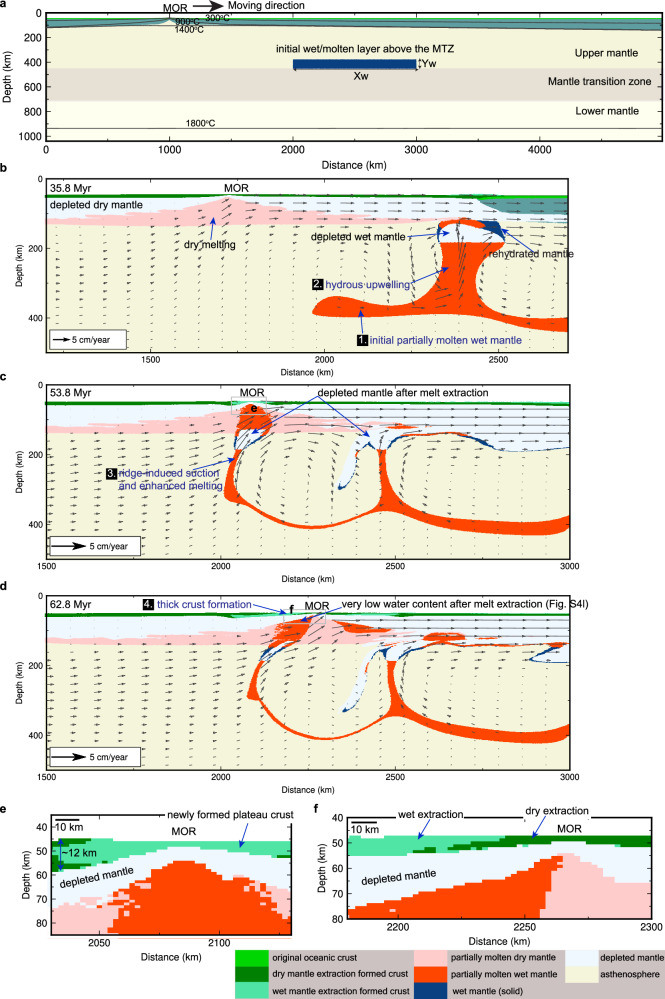
Geodynamic model of intraplate oceanic volcanism from a hydrous mantle reservoir above the MTZ. **a** The numerical model setup with a wet or partially molten mantle layer atop the MTZ. Ridge migration is shown by an arrow. (**b**–**d**) Three snapshots of geodynamic evolution for the referenced model. The evolution follows a sequential process: 1. initial partially molten wet mantle atop the MTZ; 2. hydrous upwelling develops; 3. ridge-induced suction enhances melting; 4. thick crust formation. The grey arrows indicate velocity field. The reference model uses a *Tp* of 1350 °C and a *Cw* of 0.3 wt.%. The zoom-in regions are shown to highlight the newly formed oceanic crust in (**e**, **f**). See Supplementary Video 1 for full time evolution, and Supplementary Fig. 4 for corresponding water content, viscosity, and depletion fields. MOR, Mid-Ocean Ridge; MTZ, mantle transition zone.

We conducted two-dimensional thermo-mechanical simulations using the I2VIS code^24,25^, which solves the Stokes, continuity, and energy equations. As shown in Fig. 2, the model setup consists of a stationary left plate and a right plate migrating at 4 cm yr^−1^, with the spreading ridge (centered at 1000 km) advected passively by the migrating plate (see Methods). The mantle potential temperature is fixed at 1350 °C^15^. A hydrous batch melting model^26^ is coupled with a parameterized melt extraction scheme to predict crustal thickness (see Methods). We first calibrate the model with a dry mantle, determining a melt extraction timescale (*t*_*e*_ = 0.5 ka) that reproduces a normal oceanic crustal thickness of 6.94 ± 0.83 km (Supplementary Fig. 3). Varying the full spreading rate shows that a minimum of 4 cm yr^−1^ (see Supplementary Fig. 3) is required to produce normal crustal thickness. We then constrain the melt extraction time (*t*_*e*_ = 0.5 ka) and full-spreading rate (4 cm yr^−1^) for the latter wet mantle models.

**Fig. 3:**
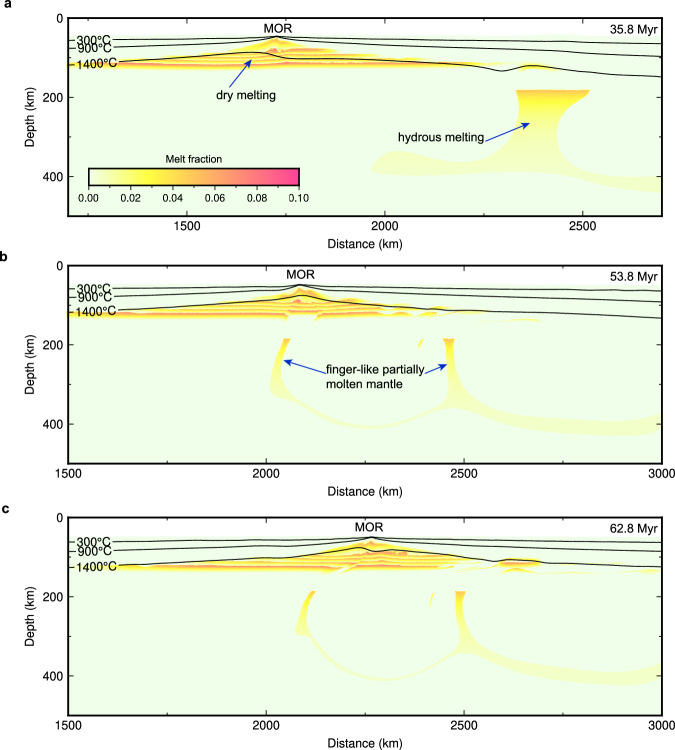
Evolution of melt fraction through time for the reference model shown in Fig. 2. Snapshot at **a** 35.8 Myr. Melting remains incipient within the Rayleigh-Taylor instability, which progressively deforms and upwells to the base of the oceanic lithosphere. **b** 53.8 Myr. The left-side instability gradually evolves into a second, narrow and slightly tilted solid mantle upwelling with a small melt fraction, nearly centered beneath the ridge axis. This process results in a double melting structure characterized by two finger-like upwellings: one located near the ridge axis and another approximately 400 km away, **c** 62.8 Myr. As the ridge migrates rightward, the left upwelling is no longer centered under the ridge axis. Black contours denote isotherms, and the position of the spreading center is marked. MOR, Mid-Ocean Ridge.

**Fig. 4:**
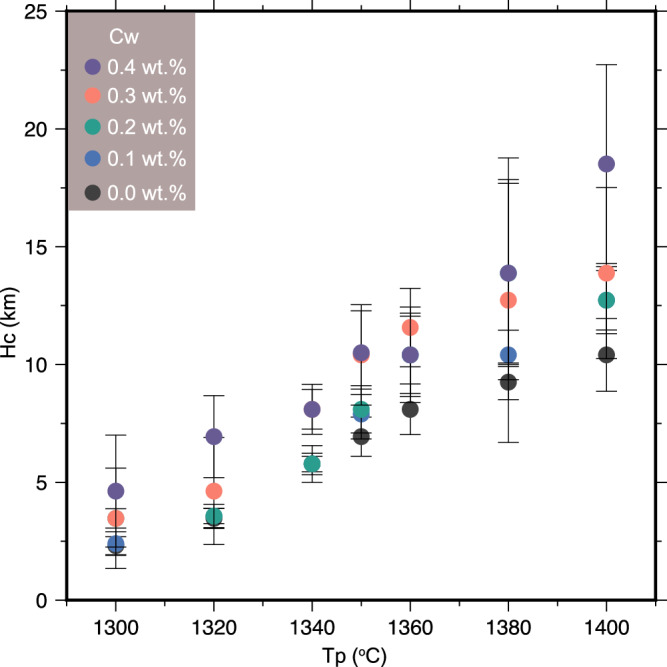
Influence of mantle potential temperature (*Tp*) and water content (*Cw*) on the oceanic mean crustal thickness *Hc.* *Hc* and error bar (1σ standard deviation) show the mean thickness and standard error of newly formed oceanic crust. The reference model (Fig. 2) assumes *Tp* = 1350 °C and *Cw* = 0.3 wt.%, with other models varying by only one parameter.

To assess how deep-mantle water heterogeneity^17,27^ modulates crustal accretion rates at Mid-Ocean Ridge (MOR), our reference wet model imposes a 1000-km-wide, 75-km-thick layer of mantle peridotite with excess water (0.3 wt.% H₂O stored in NAMs) atop the MTZ at a distance of 1000 km from the ridge axis (Fig. 2). Such a degree of hydration is sufficient to trigger mantle melting of the hydrated domain^17^. Figure 2 presents snapshots of the numerical simulation illustrating how the migrating ridge interacts with this deep-mantle anomaly. As the ridge approaches the hydrated domain, a Rayleigh-Taylor instability develops within the partially molten layer, evolving into an upwelling (radius <200 km) at 35.8 Myr, located ~1300 km from the ridge axis (Fig. 2b). Melt extraction occurs at 100–150 km depth near the edge of the MOR melting zone (triangular region) (Fig. 2b). Continued migration causes the upwelling head to flatten against the base of the lithosphere and spread laterally, driving a return flow toward the ridge axis and a secondary thin, finger-like upwelling with a limited melt fraction directly beneath the ridge center at 53.8 Myr (Figs. 2, 3, and Supplementary Fig. 5; Supplementary Video 1). At 62.8 Myr, the rightward plate motion shears hydrous melts beneath the trailing flank. In contrast, crust on the leading flank forms largely from dry melts (Fig. 2d). This asymmetry is a prediction of our model that awaits testing with future geochemical sampling of the plateau’s conjugate flanks. This simulation yields a mean crustal thickness of 10.4 ± 2.1 km formed within ca. 10 Myrs (Supplementary Video 1). Varying the water content of the hydrated domain produces mean crustal thicknesses of 7.9 ± 1.1 km for 0.1 wt.% and 10.5 ± 1.8 km for 0.4 wt.% (Fig. 4). The mean crustal thickness increases with higher mantle potential temperature (Fig. 4). The resulting crustal thickness is heterogeneous, with mean thicknesses exceeding 20 km, and locally reaching up to 25 km, comparable to that of the Azores Plateau, when 0.4 wt.% H₂O is combined with a potential temperature of 1400 °C in the simulations.

Parameter tests reveal that the size of the hydrous reservoir critically controls mantle flow patterns and the resulting magmatic output. For instance, a narrower (e.g., 500-km-wide) or thinner (e.g., 25-km-thick) wet zone yields less volcanism (Supplementary Fig. 7). Thinning the hydrous reservoir markedly displaces the secondary finger-like upwelling beneath the stationary plate, shifting it either nearer to or farther from the ridge axis (Supplementary Fig. 7). In the absence of plate motion, the hydrous partially molten layer remains stable over 100 s of Myr, even when assuming a buoyant melt atop the MTZ (Supplementary Fig. 8). Experimental studies suggest that long-term stability requires dry melt to be denser than the surrounding mantle^17,22,23^. Yet, when ridge migration is introduced, the resulting suction force can entrain partially molten rocks with melts up to 20% denser than the surrounding mantle (Supplementary Fig. 9). This occurs because the low-melt fractions characteristic of these depths ensure that viscous forces dominate and density contrasts become secondary.

### Implications for the thick crust in the Azores Plateau

While a mantle plume has long been invoked to explain the Azores Plateau, several observations suggest that additional factors, such as a hydrated mantle source and ridge-controlled dynamics, may have played a crucial role.

The plateau’s formation and evolution are intimately linked to plate boundary processes. The close match between its internal magnetic lineations and those of the surrounding seafloor since chron 6 (ca. 20 Ma) indicates it formed near a spreading ridge^5^. Its location at the Azores Triple Junction provides an efficient mechanism for focusing mantle upwelling and enhancing melt production through plate-driven flow^28^. Our model demonstrates that excess crustal production can be sustained by hydrous upwellings from the MTZ with only a minor excess temperature, consistent with the diffuse, non-age-progressive volcanism observed since ~39 Ma^7,8^.

Petrological studies of both plateau MORBs and OIBs do not require the extremely hot mantle potential temperatures (>1400 °C) predicted by a purely thermal plume. Instead, their compositions point to a mantle source enriched in water and recycled oceanic components. This is evidenced by elevated water contents, positive Nb anomalies in MORBs, and radiogenic isotope systematics (Sr-Nd-Pb-Hf) that identify recycled crustal components of varying ages (e.g., 2.5–3 Ga recycled crust beneath São Miguel and younger, dehydrated, altered crust beneath the central islands)^4,6,13,14^. These signatures are consistent with a subduction legacy stored in the MTZ, rather than a primordial, deep-mantle plume source.

The cylindrical low-velocity anomalies extending no deeper than 400 km^11^ or 250 km^29^ in tomographic images beneath the Azores Plateau do not necessarily represent deep thermal plumes anchored to the CMB. According to our numerical models, hydrous upwelling from the MTZ can produce similar features (Fig. 2 and Supplementary Fig. 6). While the presence of water can, in principle, affect the depth of the 410-km discontinuity^30^, interpreting MTZ topography beneath the Azores is complex. Seismic observations show either normal transition zone thickness^31^ or a depressed 410-km discontinuity^32^, reflecting the competing effects of temperature, composition (e.g., magnesium number), and water content^30,33^. At the ambient mantle potential temperatures assumed in our models (1350–1400 °C), the effect of water on discontinuity depth is probably minimal^33,34^.

The last stage of melting in our geodynamic model starts at ~100–150 km depth, both on- and off-axis (Supplementary Fig. 6), consistently with the initial melting pressure inferred for most Azores OIBs (~3.7–2.5 GPa)^7^ and for Azores-platform MORBs (≤3.8 GPa)^4^. Crucially, evidence for a volatile-rich mantle source extends to the plateau lavas themselves. Métrich et al.^6^ analyzed melt inclusions from Pico Island and found elevated H₂O content (up to 2.0 wt.%) and H₂O/Ce ratios (259 ± 21). Most importantly, they note that these values are similar to published ratios for submarine basalts from the Azores platform (253 ± 33)^35^, indicating that the plateau source was similarly hydrated. This is consistent with Asimow et al.^36^, who modeled that much of the crustal thickness anomaly at the Azores platform can be explained by a hydrous source (up to ~700 ppm H₂O in the mantle) with only a modest thermal anomaly (ΔT ≤ 75 °C). Beier et al.^7^ further show that Azores lavas require at least 200 ppm H₂O in the source because their melting temperatures are lower than the dry mantle solidus. Our models reproduce the thick crust observed at the Azores (e.g., 10.4 ± 2.1 km for 0.3 wt.% H₂O at 1350 °C), albeit with higher mantle source water contents than those inferred from shallow wet melting models (0.05–0.09 wt.% H₂O in the source)^6,12,36^. However, a similar crustal thickness (e.g., 12.7 ± 1.4 km) can be achieved with a lower water content (0.1 wt.%) and a modest mantle temperature (1400 °C). This discrepancy may reflect additional factors not fully accounted for in petrological models, such as the lowering effect of CO₂ on the solidus (CO₂ in Azores OIBs ca. 0.2–0.9 wt.%)^37^, or the assumption of a slightly higher local mantle potential temperatures (generally in the range 1342–1461 °C)^15^ compared to our global average (1350–1400 °C).

Our 2D models cannot resolve the complex mantle flow or along-axis lateral transport associated with the three limbs of the Azores triple junction, which may enhance upwelling through dynamic focusing^38^. Although this process alone is likely insufficient to enhance melting when adiabatic cooling is considered. In our framework, such additional mechanical suction would concentrate hydrous material from a broader volume of the MTZ into the melting region. Consequently, the presence of the triple junction may reduce the volatile threshold required in our models to achieve the observed crustal thickness. Future 3D modeling that incorporates a hydrous MTZ and full triple junction kinematics will be required to investigate the influence of source fertility and plate geometry on the formation of the Azores Plateau.

Furthermore, the scarcity of thick, plateau-like crust along most mid-ocean ridges may reflect the heterogeneous distribution of water in the MTZ. Only where a sufficiently large and highly hydrated domain interacts with a migrating ridge, as proposed for the Azores, does this process generate enough melt to produce a plateau-scale edifice. Smaller or less hydrated domains may produce more subtle anomalies, while larger domains could, under favorable tectonic conditions, generate even more massive features.

### Implication for the subduction legacy of water in the MTZ and intraplate volcanism

The MTZ represents a significant global reservoir for subduction-recycled water, a legacy of ancient tectonic processes that is central to our model for oceanic plateau formation. Plate subduction has continuously hydrated the MTZ for at least two billion years^39^, transporting water via dense hydrous magnesium silicates within descending slabs^40,41^. The MTZ minerals, wadsleyite and ringwoodite, possess exceptional water storage capacities^42^, which is attested by diamond inclusions^27^. A prominent, globally detected seismic low-velocity layer (LVL) atop the 410-km discontinuity provides compelling geophysical evidence for a thin layer of partial melt, likely produced by dehydration-induced melting of ascending hydrous MTZ material^17,20^. This partially molten layer, which may be either slightly positively or negatively buoyant (Fig. 2 and Supplementary Fig. 9), can be entrained by mantle flow, such as ridge-induced suction^43^ or far-field subduction-induced upwelling^44^. As this material rises adiabatically through the upper mantle, decompression progressively increases the melt fraction. Only when the ascending material reaches shallower depths (probably <200 km) is this melt segregated and then successively emplaced at the surface.

Hydrous upwellings sourced from the MTZ provide a coherent explanation for water-rich intraplate volcanism in regions far from active subduction zones. For instance, Bermuda^45^, East Asia^24^, eastern Australia^46^, and the South China Sea^47^ volcanism have been linked to a volatile-rich MTZ source (Fig. 1). These examples, though not all associated with plateau formation, demonstrate that hydrous MTZ upwelling is a plausible and potentially widespread process.

Although the Azores are now distant from active subduction, their underlying mantle was likely hydrated during the prolonged subduction of the Farallon plate since at least 140 Ma^48^. This inference is strongly supported by recent observations. The Farallon slab was vast, hydration of the overlying MTZ was probably heterogeneous, controlled by variations in slab temperature, composition, and structure during dehydration^40,41^, as well as by the amount of oceanic plate hydration depending on several processes occurring at the surface^49^. Consistent with this view, seismic low-velocity anomalies and intraplate volcanism in eastern North America, including Bermuda, have been attributed to hydrous melting originating from the MTZ, likely due to Farallon slab dehydration^45,50,51^. Further support comes from the equatorial Atlantic, where high-resolution seismic imaging reveals significant MTZ thinning, indicative of ridge suction drawing material upwards^43^, coincident with the eruption of water-rich basalts^52^. Recent geochemical studies have also confirmed that MORBs in various locations can be water-rich, some of them with evidence for recycled MTZ material contributing to the magmatism^45,47,53,54^.

Our model offers a valid alternative to the canonical mantle plume hypothesis for reevaluating the genesis of oceanic plateaus. For instance, the vast Ontong Java Nui Plateau may represent an end-member case of this mechanism, in which a particularly extensive, water-rich domain in the MTZ interacted with a triple junction (i.e., Farallon-Phoenix-Pacific)^55^. The triple junction geometry would enhance melt focusing and extraction, potentially explaining the exceptional volume of this plateau. The presence of HIMU-like and FOZO-like signatures in lavas may lend credence to the presence of a hydrous mantle source^56^. Similarly, our model demonstrates that hydrous MTZ upwelling can sustain spatially and temporally distributed volcanism over ~10 Myr timescales beneath the Azores, consistent with the observed duration of platform construction^28^. This mechanism may offer a framework for reevaluating the genesis of other oceanic plateaus (such as Galápagos and Amsterdam-St Paul), particularly those formed in near-ridge settings, although detailed testing awaits comparable geochemical and geophysical datasets.

We emphasize that hydrous MTZ upwelling is not proposed as a replacement for mantle plumes in all intraplate settings. Localities such as Hawaii and Iceland exhibit clear evidence for deep-rooted thermal anomalies, including excess mantle temperatures, age-progressive tracks, and lower-mantle seismic structures. Instead, our mechanism is offered as an alternative process that may operate in specific tectonic contexts, particularly where a migrating ridge interacts with a localized, hydrated MTZ domain. The Azores serve as a typical example of such a setting, where observations (modest thermal excess, lack of age progression, and volatile-rich signatures) are difficult to reconcile with a classical plume but are naturally explained by hydrous mantle upwelling.

This process also offers new insights into resolving the longstanding δD paradox^12^. Although subducted slabs are expected to be deuterium-depleted, enriched mantle sources often exhibit anomalously heavy δD values. Such values can arise from the upward transport of water via hydrous MTZ upwellings. In this process, water-rich MTZ minerals (wadsleyite, ringwoodite) may release free fluids or undergo hydrous melting upon crossing the 410-km discontinuity, depending on the water content^17^. These water-bearing melts or fluids remain within the upwelling material and, as it rises, metasomatize the surrounding upper mantle, effectively transferring water from the MTZ to shallower depths. The mechanism may also explain the coexistence of recycled slab signatures with near primordial ³He/⁴He ratios in both OIBs and MORBs. High ³He/⁴He ratios (up to 18.4 R_A_) observed in some Azores lavas have been interpreted as evidence of a lower mantle plume^57–59^. However, super‑deep diamonds from the MTZ, carrying a wide-ranging signature in Sr-Pb-C isotopes coupled to extremely high and variable ³He/⁴He ratios (0.7–49.9), point to infiltration of a primordial, less‑degassed helium-bearing reservoir mixing with recycled slab material within the MTZ^60^. In our model, hydrous upwellings originating from the MTZ, driven by dehydration melting and ridge suction, not deep thermal buoyancy, can sample this mixed reservoir and transport both signatures to the surface. This offers an alternative to classical plume models: the same geochemical observations are explained by a different source location (MTZ rather than CMB) and mobilization mechanism (hydration-driven upwelling rather than thermal buoyancy), without requiring a deep, hot plume anchored to the CMB. It may also account for the widespread contamination of large-scale upper mantle domains by subduction-related fluid signature (DUPAL and South Atlantic domains)^61–63^, thereby linking deep-Earth volatile cycling to shallow upper mantle processes and surface magmatism.

## Methods

### Numerical modeling approach

The 2D thermomechanical numerical code I2VIS^24,25^ solves the conservation equations of mass, momentum, and energy using a finite-difference method, marker-in-cell technique on a staggered grid:

1 $$\frac{\partial {v}_{i}}{\partial {x}_{i}}=0$$

2 $$-\frac{\partial P}{\partial {x}_{i}}+\frac{\partial {\tau }_{{ij}}}{\partial {x}_{j}}+\rho {g}_{i}=0$$

3 $$\rho {c}_{p}\left(\frac{\partial T}{\partial t}+{v}_{i}\frac{\partial T}{\partial {x}_{i}}\right)=\frac{\partial }{\partial {x}_{i}}\left(k\frac{\partial T}{\partial {x}_{i}}\right)+{H}_{r}+{H}_{s}+{H}_{a}$$

In these formulations, ${v}_{i}$ denotes velocity, ${x}_{i}$ the spatial coordinate, $P$ the pressure, and ${\tau }_{{ij}}={\sigma }_{{ij}}+P{\delta }_{{ij}}$ the deviatoric stress tensor with ${\sigma }_{{ij}}$ representing the total stress tensor. Additional variables include $\rho$ (density), $g$ (gravitational acceleration), ${c}_{p}$ (isobaric specific heat capacity), $T$ (temperature), $t$ (time), $k$ (thermal conductivity), and the heat sources ${H}_{r}$ (radioactive heating), ${H}_{s}$ (shear heating) and ${H}_{a}$ (adiabatic heating).

A visco-plastic rheological model is adopted to describe rock deformation. Viscous flow in the mantle is governed by diffusion and dislocation creep^64^. The presence of water or melt reduces the effective viscosity, with weakening implemented as follows:

4 $${\eta }_{{wet}/{melt}}={\eta }_{{dry}}{\left(\frac{{C}_{w}}{{C}_{w0}}\right)}^{-\frac{r}{n}}exp \left(-\frac{\alpha \phi }{n}\right)$$

5 $${\eta }_{{diff}/{disl}}=\frac{1}{2}G{A}_{d}^{-1/n}{\left(\frac{b}{d}\right)}^{-m/n}{\dot{\varepsilon }}_{{II}}^{1/n-1}\exp \left(\frac{E+{PV}}{{nRT}}\right)$$

6 $$\frac{1}{{\eta }_{{dry}}}=\frac{1}{{\eta }_{{diff}}}+\frac{1}{{\eta }_{{disl}}}$$

where ${C}_{w}$ and ${C}_{w0}$ denote the water content in the rock and the reference water content, respectively; and $r$ (water fugacity exponent), $n$ (stress exponent), $\alpha$ (melt-weakening factor) are the experimentally derived parameters^64,65^ (Supplementary Table 1). ${\eta }_{{dry}}$ is the viscosity of dry mantle, whereas ${\eta }_{{wet}/{melt}}$ represents viscosity when water and/or melt is present. *A*_*d*_ is the prefactor, *G* the shear modulus, *b* the Burgers vector, and *d* the grain size, $m$ the grain size exponent, *E* the activation energy, *V* the activation volume, and *R* the gas constant. The effective viscosity of dry mantle rock is expressed as the harmonic mean of the viscosities for diffusion creep (${\eta }_{{diff}}$) and dislocation creep (${\eta }_{{diff}}$). Parameter values are listed in Supplementary Table 1.

We also consider the solid-solid phase changes for the basalt and mantle derived from PERPLE_X^66^. Therefore, the phase changes at the mantle transition zone interfaces, i.e., 410 km and 660 km, have been considered. The numerical approaches have been detailed elsewhere^24,25^.

### Model setup

The model domain is a 2D rectangular region measuring 4998 km × 1040 km (Fig. 2a), discretized using 715 × 261 Eulerian nodes. It includes two 50-Myr-old oceanic plates separated by a mid-ocean ridge, initially located 1000 km from the model origin. The left plate is stationary (*V*_*LP*_ = 0 cm yr^−1^; horizontal velocities are positive when oriented rightward), and the right-side plate is migrating at *V*_*RP*_ = 4 cm yr^−1^, therefore the half-spreading rate is *V*_*SR*_ = (*V*_*RP*_ + *V*_*LP*_)/2 = 2 cm yr^−1^ and with full-spreading rate *V*_*FS*_ = *V*_*RP*_ *- V*_*LP*_ = 4 cm yr^−1^. The spreading ridge migrates rightward at a rate of *V*_*SR*_ = 2 cm yr^−1^, with open lateral boundaries to permit mass flux and a 40-km-thick “sticky air” layer approximating an internal free surface. Temperatures are fixed at 0 °C and 1850 °C at the top and bottom, respectively. The lateral boundaries are treated as insulating (zero heat flux). A mantle potential temperature of 1350 °C is used, and the underlying mantle has an adiabatic gradient of 0.5 °C km^−1^. Additional simulations explored alternative potential temperatures. To examine the effect of hydration on thick crust formation, a water-rich mantle domain (0.3 wt.%) is placed atop the MTZ between 2000 km and 3000 km in the x-direction. Such a water enrichment represents a moderate approximation of present-day values^18,67,68^ (Fig. 1a). Model sensitivity tests varied the initial water content and the size of the wet zone.

### Mantle melting and melt extraction

Here, we adopt the mantle melting parameterization of ref. ^26^ for the upper mantle. To isolate the effects of hydrous melting in the upper mantle region, melting below 410 km is suppressed, because hydrous partial melting at greater depths remains poorly constrained. The parameterization is calibrated for pressures <8 GPa; therefore, we linearly extrapolate the solidus curves (the quadratic polynomials) to 410 km. The latent heat of mantle melting is accounted implicitly in the formulation.

The melt extraction timescale, *t*_*e*_ (eq. 14 in ref. ^24^), parametrizes the efficiency of melt segregation from the solid matrix, which is controlled by melt fraction, melt viscosity, density contrast, and mantle viscosity. The melt extraction timescale *t*_*e*_ is a crucial proxy for melt extraction and the newly formed crust. Melt extraction is considered efficient when the timescale of the partially molten material is shorter than the reference timescale. Larger reference *t*_*e*_ values correspond to more efficient melt extraction, whereas smaller values result in less extraction. Consequently, decreasing the reference *t*_*e*_ produces a thinner crust, whereas increasing the reference *t*_*e*_ yields a thicker crust. We adopt a reference *t*_*e*_ of 500 years, which yields a realistic oceanic crustal thickness (6.94 ± 0.83 km) for a dry mantle (Supplementary Fig. 3). Extracted melt is assumed to be instantaneously emplaced at the surface to form volcanic rocks^69,70^. In our models, the hydrated layer atop the MTZ consists of mantle peridotite with excess water (0.1–0.4 wt.% H₂O) stored in nominally anhydrous minerals. At its initial depth (~410 km), this material experiences only minor partial melting (melt fraction ≤1%) due to the presence of water. At such low fractions, the melt remains interconnected but cannot escape the solid matrix because of high ambient viscosity and low permeability. The partially molten material is therefore retained and remains buoyant relative to the dry mantle. As ridge-induced suction entrains this material upward, decompression increases the melt fraction. Only when the partially molten rocks reach our parameterized melt extraction scheme does melt segregation occur. This typically occurs at depths <200 km. Upon partial melting and extraction, the water is partitioned into the extracted melt and water in the residual peridotite as:

7 $${C}_{w}^{{melt}}=\frac{{C}_{w}}{\phi \left(1-D\right)+D}$$

8 $${C}_{w}^{{res}}=\frac{{C}_{w}-\phi {C}_{w}^{{melt}}}{\left(1-\phi \right)}$$

## Supplementary information

Supplementary Information

Description of Additional Supplementary Files

Supplementary Video 1

Transparent Peer Review file

## Data Availability

The data used in Fig. 1 are from corresponding references (Zhou et al., 2022; Zhou et al., 2023). The model output for the reference model is available in FigShare^71^ (10.6084/m9.figshare.32100925).

## Code availability

Requests about the numerical code I2VIS should be addressed to the main code developer, T. Gerya (taras.gerya@erdw.ethz.ch), or the corresponding author. Figures are produced by the open-source code Generic Mapping Tools (https://www.generic-mapping-tools.org, Version 6.5.0).

## References

[1] Richards, M. A., Duncan, R. A. & Courtillot, V. E. Flood basalts and hot-spot tracks: plume heads and tails. *Science* **246**, 103–107 (1989). 17837768 10.1126/science.246.4926.103

[2] Coffin, M. F. et al. Kerguelen hotspot magma output since 130 Ma. *J. Pet.* **43**, 1121–1137 (2002).

[3] Sager, W. W., Sano, T. & Geldmacher, J. Formation and evolution of Shatsky Rise oceanic plateau: Insights from IODP Expedition 324 and recent geophysical cruises. *Earth-Sci. Rev.* **159**, 306–336 (2016).

[4] Asimow, P. D. & Langmuir, C. H. The importance of water to oceanic mantle melting regimes. *Nature* **421**, 815–820 (2003). 12594505 10.1038/nature01429

[5] Gente, P., Dyment, J., Maia, M. & Goslin, J. Interaction between the Mid-Atlantic Ridge and the Azores hot spot during the last 85 Myr: Emplacement and rifting of the hot spot-derived plateaus. *Geochem. Geophys. Geosyst.* **4**, 10.1029/2003GC000527 (2003).

[6] Métrich, N. et al. Is the ‘Azores hotspot’a wetspot? Insights from the geochemistry of fluid and melt inclusions in olivine of Pico basalts. *J. Pet.* **55**, 377–393 (2014).

[7] Beier, C., Haase, K. M. & Turner, S. P. Conditions of melting beneath the Azores. *Lithos* **144**, 1–11 (2012).

[8] Beier, C., Haase, K,M.& Abouchami, W. Geochemical and geochronological constraints on the evolution of the Azores Plateau. In: *The Origin, Evolution, and Environmental Impact of Oceanic Large Igneous Provinces* (eds Neal CR, Sager WW, Sano T, Erba E). Geological Society of America (2015).

[9] Vogt, P.R., Jung, W.-Y. The “Azores Geosyndrome” and plate tectonics: Research history, synthesis, and unsolved puzzles. In: *Volcanoes of the Azores: Revealing the geological secrets of the central Northern Atlantic Islands*. Springer (2018).

[10] Marques, F. O., Balázs, A., Gerya, T. V. & Hildenbrand, A. Dynamics and evolution of the Azores Triple Junction and its relation to pre-existing major tectonic structures. *Commun. Earth Environ.* **6**, 303 (2025).

[11] Yang, T., Shen, Y., van der Lee, S., Solomon, S. C. & Hung, S.-H. Upper mantle structure beneath the Azores hotspot from finite-frequency seismic tomography. *Earth Planet Sci. Lett.* **250**, 11–26 (2006).

[12] Dixon, J. E. et al. Light stable isotopic compositions of enriched mantle sources: Resolving the dehydration paradox. *Geochem, Geophys, Geosyst,* **18**, 3801–3839 (2017).

[13] Béguelin, P., Bizimis, M., McIntosh, E. C., Cousens, B. & Clague, D. A. Sources vs processes: Unraveling the compositional heterogeneity of rejuvenated-type Hawaiian magmas. *Earth Planet Sci. Lett.* **514**, 119–129 (2019).

[14] Marzoli, A. et al. Time-dependent evolution and source heterogeneities of Ocean Island Basalts From a Weak Plume, São Jorge, Azores. *J. Petrol.* **65**, egae122 (2024).

[15] Herzberg, C. & Gazel, E. Petrological evidence for secular cooling in mantle plumes. *Nature* **458**, 619 (2009). 19340079 10.1038/nature07857

[16] Kohlstedt, D., Keppler, H. & Rubie, D. Solubility of water in the α, β and γ phases of (Mg, Fe) 2SiO4. *Contrib. Miner. Pet.* **123**, 345–357 (1996).

[17] Bercovici, D. & Karato, S. -i Whole-mantle convection and the transition-zone water filter. *Nature* **425**, 39–44 (2003). 12955133 10.1038/nature01918

[18] Karato, S. -i, Karki, B. & Park, J. Deep mantle melting, global water circulation and its implications for the stability of the ocean mass. *Prog. Earth Planet Sci.* **7**, 76 (2020).

[19] Revenaugh, J. & Sipkin, S. Seismic evidence for silicate melt atop the 410-km mantle discontinuity. *Nature* **369**, 474–476 (1994).

[20] Tauzin, B., Debayle, E. & Wittlinger, G. Seismic evidence for a global low-velocity layer within the Earth’s upper mantle. *Nat. Geosci.* **3**, 718 (2010).

[21] Wang, X. et al. Distinct slab interfaces imaged within the mantle transition zone. *Nat. Geosci.* **13**, 822–827 (2020).

[22] Matsukage, K. N., Jing, Z. & Karato, S. -i Density of hydrous silicate melt at the conditions of Earth’s deep upper mantle. *Nature* **438**, 488–491 (2005). 16306990 10.1038/nature04241

[23] Sakamaki, T., Suzuki, A. & Ohtani, E. Stability of hydrous melt at the base of the Earth’s upper mantle. *Nature* **439**, 192–194 (2006). 16407950 10.1038/nature04352

[24] Yang, J. & Faccenda, M. Intraplate volcanism originating from upwelling hydrous mantle transition zone. *Nature* **579**, 88–91 (2020). 32103183 10.1038/s41586-020-2045-y

[25] Gerya,T. V. & Yuen, D. A. Characteristics-based marker-in-cell method with conservative finite-differences schemes for modeling geological flows with strongly variable transport properties. *Phys. Earth Planet In.* **140**, 293–318 (2003).

[26] Katz, R. F., Spiegelman, M. & Langmuir, C. H. A new parameterization of hydrous mantle melting. *Geochem. Geophys. Geosyst*. **4**, 1073 (2003).

[27] Pearson, D. G. et al. Hydrous mantle transition zone indicated by ringwoodite included within diamond. *Nature* **507**, 221–224 (2014). 24622201 10.1038/nature13080

[28] Cannat, M. et al. Mid-Atlantic Ridge–Azores hotspot interactions: along-axis migration of a hotspot-derived event of enhanced magmatism 10 to 4 Ma ago. *Earth Planet Sci. Lett.* **173**, 257–269 (1999).

[29] Silveira, G. et al. Azores hotspot signature in the upper mantle. *J. Volcano Geotherm. Res*. **156**, 23–34 (2006).

[30] Smyth, J. R. & Jacobsen, S. D. Nominally anhydrous minerals and Earth’s deep water cycle. *Geophys. Monogr. -Am. Geophys. Union* **168**, 1 (2006).

[31] Silveira, G. et al. Stratification of the Earth beneath the Azores from P and S receiver functions. *Earth Planet Sci. Lett.* **299**, 91–103 (2010).

[32] Saki, M., Thomas, C., Nippress, S. E. J. & Lessing, S. Topography of upper mantle seismic discontinuities beneath the North Atlantic: The Azores, Canary and Cape Verde plumes. *Earth Planet Sci. Lett.* **409**, 193–202 (2015).

[33] Frost, D. J. The upper mantle and transition zone. *Elements* **4**, 171–176 (2008).

[34] Litasov, K. D. & Ohtani, E. Effect of water on the phase relations in Earth’s mantle and deep water cycle. *Geol. Soc. Am. Spec. Pap.* **421**, 115–156 (2007).

[35] Dixon, J. E., Leist, L., Langmuir, C. & Schilling, J.-G. Recycled dehydrated lithosphere observed in plume-influenced mid-ocean-ridge basalt. *Nature* **420**, 385–389 (2002). 12459776 10.1038/nature01215

[36] Asimow, P.D., Dixon, J., Langmuir, C. A hydrous melting and fractionation model for mid-ocean ridge basalts: Application to the Mid-Atlantic Ridge near the Azores. *Geochem. Geophys. Geosyst.* **5**, (2004).

[37] van Gerve, T. D. et al. The origin and differentiation of CO_2_-rich primary melts in ocean island volcanoes: integrating 3D X-Ray Tomography with chemical microanalysis of olivine-hosted melt inclusions from pico (Azores). *J. Pet.* **65**, egae006 (2024).

[38] Georgen, J. E. Mantle flow and melting beneath oceanic ridge–ridge–ridge triple junctions. *Earth Planet Sci. Lett.* **270**, 231–240 (2008).

[39] Wan, B. et al. Seismological evidence for the earliest global subduction network at 2 Ga ago. *Sci. Adv.* **6**, eabc5491 (2020). 32821847 10.1126/sciadv.abc5491 PMC7406333

[40] van Keken, P. E., Hacker, B. R., Syracuse, E. M. & Abers, G. A. Subduction factory: 4. Depth-dependent flux of H_2_O from subducting slabs worldwide. *J. Geophys. Res: Solid Earth* **116**, 10.1029/2010JB007922 (2011).

[41] Yang, J. & Faccenda, M. On the dynamics of water transportation and magmatism in the mid-mantle. *J. Geophys Res: Solid Earth* **128**, e2023JB026469 (2023).

[42] Kohlstedt, D. L., Keppler, H. & Rubie, D. C. Solubility of water in the α, β, and γ phases of (Mg, Fe)2SiO4. *Contrib. Miner. Pet.* **123**, 345–357 (1996).

[43] Agius, M. R., Rychert, C. A., Harmon, N., Tharimena, S. & Kendall, J. M. A thin mantle transition zone beneath the equatorial Mid-Atlantic Ridge. *Nature* **589**, 562–566 (2021). 33505039 10.1038/s41586-020-03139-x

[44] Yang, J. et al. The origin and fate of subslab partial melts at convergent margins. *Natl. Sci Rev.* **12**, nwaf314 (2025).

[45] Mazza, S. E. et al. Sampling the volatile-rich transition zone beneath Bermuda. *Nature* **569**, 398–403 (2019). 31092940 10.1038/s41586-019-1183-6

[46] Mather, B. R. et al. Intraplate volcanism triggered by bursts in slab flux. *Sci. Adv.* **6**, eabd0953 (2020). 33328233 10.1126/sciadv.abd0953 PMC7744089

[47] Qian, S.-P., Gazel, E. & Wang, J.-H. Mantle transition zone water triggers lithospheric weakening and spreading. *Geology* **53**, 461–466 (2025).

[48] Shephard, G. E. et al. Circum-Arctic mantle structure and long-wavelength topography since the Jurassic. *J. Geophys Res: Solid Earth* **119**, 7889–7908 (2014).

[49] Faccenda, M. Water in the slab: A trilogy. *Tectonophysics* **614**, 1–30 (2014).

[50] van der Lee, S., Regenauer-Lieb, K. & Yuen, D. A. The role of water in connecting past and future episodes of subduction. *Earth Planet Sci. Lett.* **273**, 15–27 (2008).

[51] Liu, S., King, S. D., Long, M. D., Benoit, M. H. & Aragon, J. C. Receiver Function Analysis Reveals Lateral Variations in Temperature and Water Content in the Mantle Transition Zone Beneath Eastern North America. *Geophys. Res Lett.* **50**, e2022GL101965 (2023).

[52] Ligi, M., Bonatti, E., Cipriani, A. & Ottolini, L. Water-rich basalts at mid-ocean-ridge cold spots. *Nature* **434**, 66–69 (2005). 15744299 10.1038/nature03264

[53] Yang, A. Y. et al. A subduction influence on ocean ridge basalts outside the Pacific subduction shield. *Nat. Commun.* **12**, 4757 (2021). 34362917 10.1038/s41467-021-25027-2 PMC8346520

[54] Zhou, J. et al. A machine learning based-approach to predict the water content of mid-ocean ridge basalts. *Geochem Geophys Geosyst.* **24**, e2023GC010984 (2023).

[55] Konter, J. G. et al. Pacific hotspots reveal a Louisville–Ontong Java Nui tectonic link. *Nature* **641**, 388–394 (2025). 10.1038/s41586-025-08889-0 40307546

[56] Tejada, M. L. G. et al. New evidence for the Ontong Java Nui hypothesis. *Sci. Rep.* **13**, 8486 (2023). 37231104 10.1038/s41598-023-33724-9 PMC10213030

[57] Madureira, P., Moreira, M., Mata, J. & Allègre, C. J. Primitive neon isotopes in Terceira Island (Azores archipelago). *Earth Planet Sci. Lett.* **233**, 429–440 (2005).

[58] Madureira, P. et al. Helium isotope systematics in the vicinity of the Azores triple junction: Constraints on the Azores geodynamics. *Chem. Geol.* **372**, 62–71 (2014).

[59] Moreira MA, Madureira P, Mata J. Noble gas constraints on the origin of the Azores Hotspot. In: Volcanoes of the Azores: *Revealing the geological secrets of the central northern Atlantic islands*. Springer (2018).

[60] Timmerman, S. et al. Primordial and recycled helium isotope signatures in the mantle transition zone. *Science* **365**, 692–694 (2019). 31416962 10.1126/science.aax5293

[61] Le Roux, P. et al. Mantle heterogeneity beneath the southern Mid-Atlantic Ridge: trace element evidence for contamination of ambient asthenospheric mantle. *Earth Planet Sci. Lett.* **203**, 479–498 (2002).

[62] Meyzen, C. M, et al. New insights into the origin and distribution of the DUPAL isotope anomaly in the Indian Ocean mantle from MORB of the Southwest Indian Ridge. *Geochem. Geophys. Geosyst.* **6**, (2005).

[63] Meyzen, C. M. et al. Isotopic portrayal of the Earth’s upper mantle flow field. *Nature* **447**, 1069–1074 (2007). 17597754 10.1038/nature05920

[64] Karato, S. -i. & Wu, P. Rheology of the upper mantle: A synthesis. *Science* **260**, 771–778 (1993). 17746109 10.1126/science.260.5109.771

[65] Hirth, G. & Kohlstedt, D. L. Water in the oceanic upper mantle: implications for rheology, melt extraction and the evolution of the lithosphere. *Earth Planet Sci. Lett.* **144**, 93–108 (1996).

[66] Connolly, J. Computation of phase equilibria by linear programming: a tool for geodynamic modeling and its application to subduction zone decarbonation. *Earth Planet Sci. Lett.* **236**, 524–541 (2005).

[67] Zhou, W.-Y. et al. Constraining composition and temperature variations in the mantle transition zone. *Nat. Commun.* **13**, 1094 (2022). 35232983 10.1038/s41467-022-28709-7 PMC8888558

[68] Wang, W., Walter, M. J., Peng, Y., Redfern, S. & Wu, Z. Constraining olivine abundance and water content of the mantle at the 410-km discontinuity from the elasticity of olivine and wadsleyite. *Earth Planet Sci. Lett.* **519**, 1–11 (2019).

[69] Yang, J. et al. Slab-triggered wet upwellings produce large volumes of melt: Insights into the destruction of the North China Craton. *Tectonophysics* **746**, 266–279 (2018).

[70] Sizova, E., Gerya, T., Brown, M. & Perchuk, L. Subduction styles in the Precambrian: Insight from numerical experiments. *Lithos* **116**, 209–229 (2010).

[71] Yang, J., Faccenda, M., Meyzen, C.& Marzoli, A. Zhao L. MOR-Azores. 10.6084/m9.figshare.32100925 (2026).

