# Supplementary material for: Subduction legacies in the mantle transition zone modulate intraplate oceanic volcanism
Source: Nat Commun. 2026 May 18;17:6566. doi: 10.1038/s41467-026-73403-7 (PMC13381961; doi:10.1038/s41467-026-73403-7)
Supplement: Supplementary file 1 — Supplementary Information [file 41467_2026_73403_MOESM1_ESM.pdf]

Supplementary Information

# Subduction legacies in the mantle transition zone modulate intraplate oceanic volcanism

Jianfeng Yang[1,2*], Manuele Faccenda[3], Christine M. Meyzen[3], Andrea Marzoli[4], Liang Zhao[5,2]

[1]State Key Laboratory of Lithospheric and Environmental Coevolution, Institute of Geology and Geophysics, Chinese Academy of Sciences, Beijing 100029, China

[2]College of Earth and Planetary Sciences, University of Chinese Academy of Sciences, Beijing 100049, China

[3]Dipartimento di Geoscienze, Università di Padova, Padova 35131, Italy

[4]Dipartimento Territorio e Sistemi Agro-Forestali, Università di Padova, Padova 35020, Italy

[5]Key Laboratory of Deep Petroleum Intelligent Exploration and Development, Institute of Geology and Geophysics, Chinese Academy of Sciences, Beijing 100029, China

*yangjf@mail.iggcas.ac.cn

## Text S1 Hypothetical interaction between a deep thermal plume and a hydrated MTZ

It is worth considering how a deep, hot mantle plume (originating from the core-mantle boundary) might interact with a pre-existing hydrated domain in the mantle transition zone. While this scenario is not the focus of our study, we briefly discuss its potential implications here.

If a deep thermal plume were to rise and encounter a hydrated MTZ, several effects could be anticipated. The excess heat might further reduce the viscosity of the already weakened, water-rich MTZ material, potentially enhancing upwelling rates and increasing the total volume of melt generated. Higher temperatures would also increase melt fractions both within the MTZ (during dehydration melting) and during subsequent decompression melting in the upper mantle. Geochemically, the resulting magmas might record mixing between the deep plume component (such as high $^{3}He/^{4}He$) and the hydrated, recycled component stored in the MTZ.

For the Azores specifically, the available observations do not require such a hybrid scenario. The mantle potential temperatures inferred for Azores lavas (≤1490ºC; ΔT ≤75ºC) are modest compared to typical plume estimates[1, 2, 3, 4], and the absence of clear age-progressive volcanism and consistently high $^3He/^4He$ ratios could be interpreted as evidence against a dominant deep plume contribution. However, these observations do not definitively rule out a deep thermal component, which could be partially masked by interactions with the hydrated MTZ or by subsequent mixing during melt extraction.

Our primary conclusion is that the contribution of a deep-rooted thermal plume, if any, is likely minimal to explain the Azores Plateau. The data are consistent with a model where volcanism is driven primarily by hydrous upwelling from the MTZ. Whether a deep plume component is entirely absent or simply subordinate remains an open question that future studies by integrating geodynamic modeling with more detailed geochemical tracers may help resolve.

**Text S2 Model limitations and future directions**

Direct geochemical data from the Azores Plateau lavas themselves remain limited. While on-axis MORB show elevated $H_2O$ ratios consistent with a hydrated source[2, 5], and OIB from Pico Island exhibit similar enrichments that match reported platform values[6], no systematic $H_2O$ measurements yet exist for the off-axis plateau basalts. Our model predicts that these lavas should also record elevated water contents, reflecting their origin from hydrous MTZ upwellings. Testing this prediction will require future ocean drilling and geochemical analyses of plateau samples.

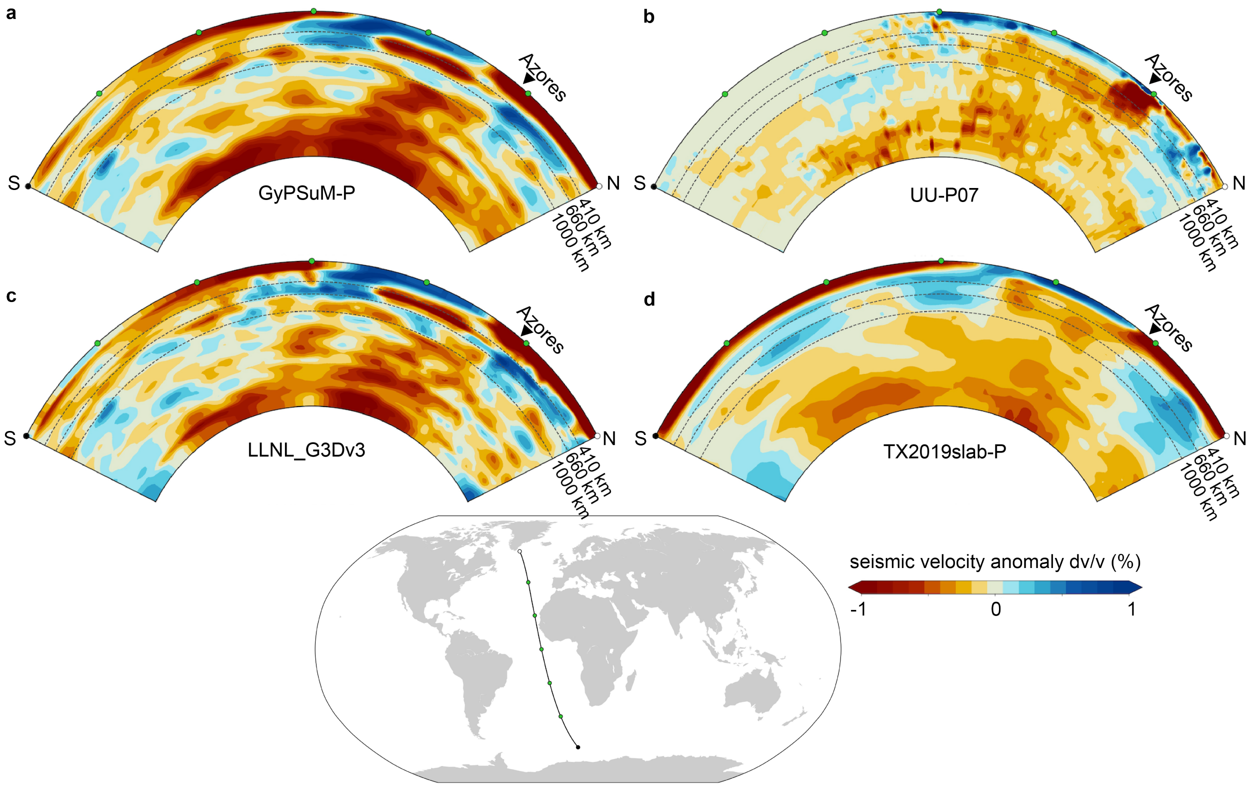


**Fig. S1 | Seismic tomography models beneath the Atlantic**. Whole mantle P-wave models of **a**, GyPSuM-P, **b**, UUP07, **c**, LLNL_G3Dv3, and **d**, TX2019slab-P from the surface to the core-mantle boundary (adopted from SubMachine[7]). Three dashed lines mark the depths at 410 km, 660 km, and 1000 km, respectively. The Azores are indicated.

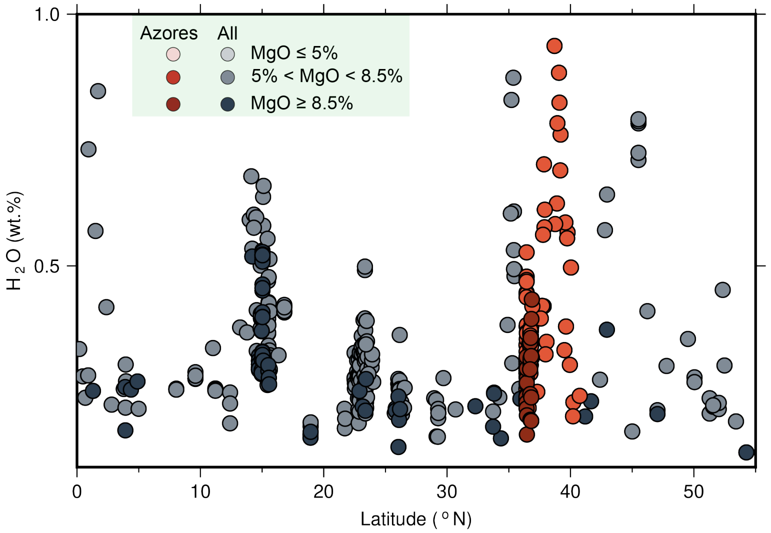


**Fig. S2 | Latitudinal profile of water contents (wt.%) in MORB along the Mid-Atlantic Ridge.**

The data is taken from ref[8] as shown in Fig. 1b. MgO content is indicated by the color scale.

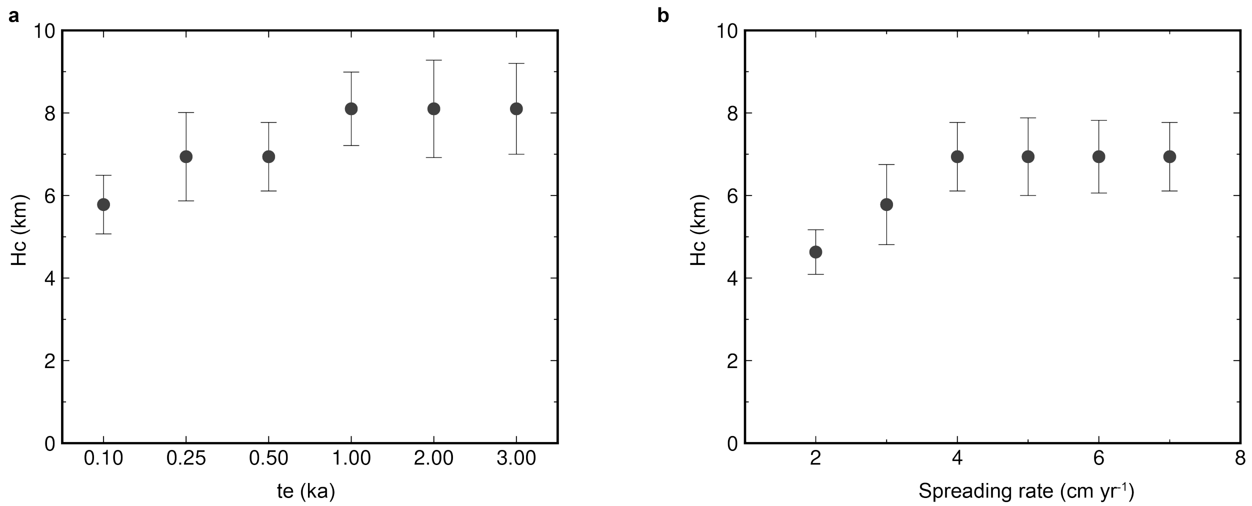


**Fig. S3 | Calibration tests for model parameters to reproduce mean oceanic crustal thickness Hc (error bar 1σ standard deviation), assuming a uniform mantle potential temperature of 1350ºC and a dry MTZ source.** Influence of **a**, melt extraction timescale ($t_e$), and **b**, spreading rate on the thickness of newly formed oceanic crust. The left plate is fixed, and the right-side plate is moving at a rate of 4 cm $yr^{-1}$ for the models in **a**, while $t_e$ = 0.5 ka in **b**.

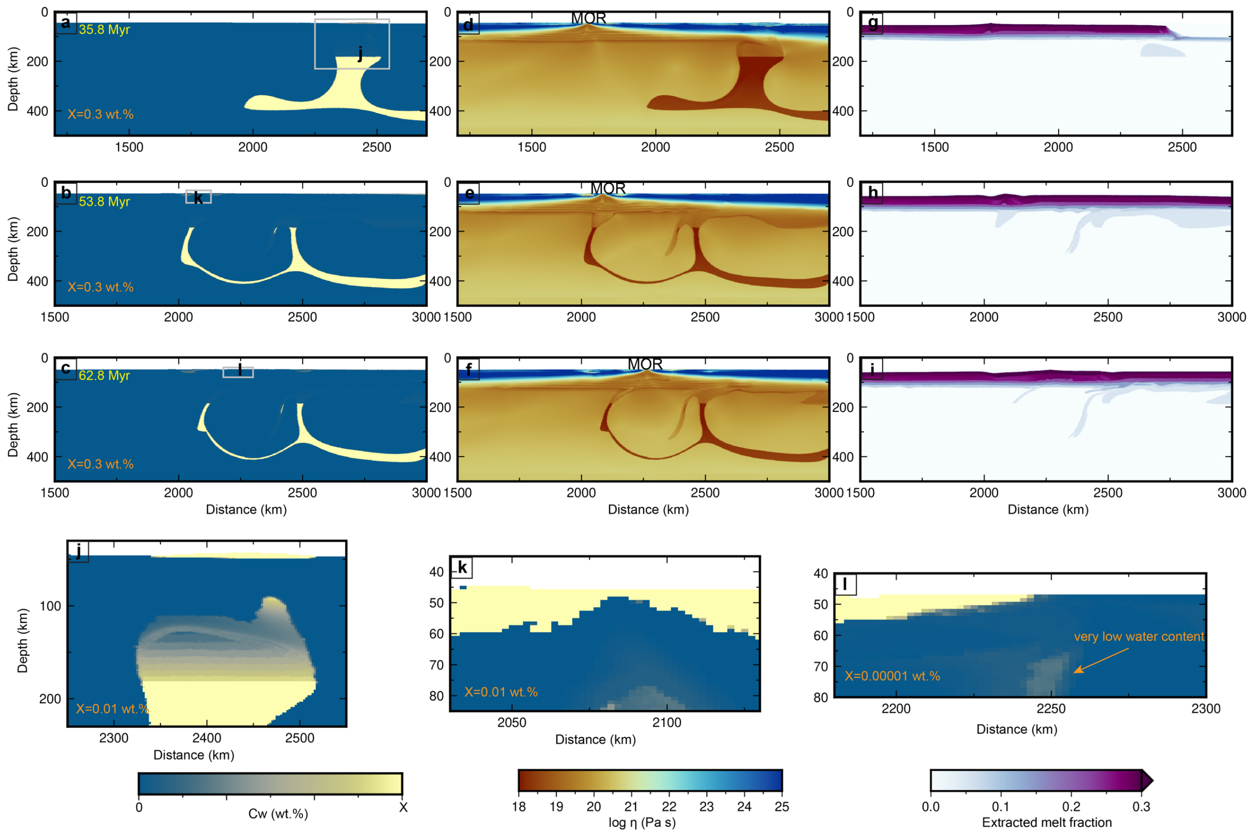


**Fig. S4 | Model evolution of the water content, viscosity, and extracted melt fraction fields for the reference model.** (**a,b,c**), Water content. (**d,e,f**), Effective viscosity. (**g,h,i**), Extracted melt fraction. (**j,k,l**) The zoom-in regions (grey boxes in **a,b,c**) show the water contents, note the different color-scale X for improving visibility.

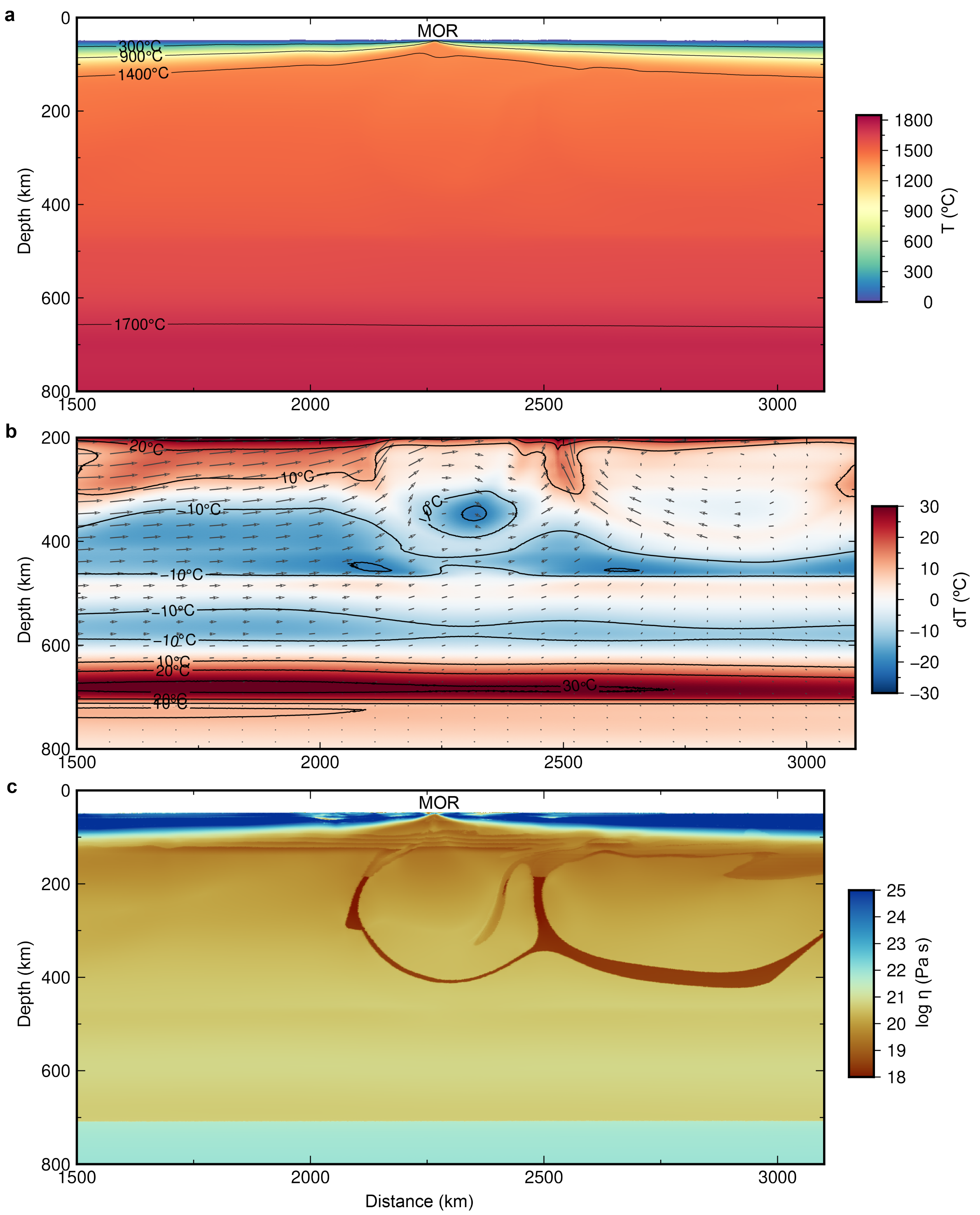


**Fig. S5 | Temperature and viscosity fields at 62.8 Myr for the final stage of the reference model.** **a**, Temperature field. **b**, The temperature anomalies relative to the temperature in the leftmost column. Arrows indicate the mantle flow field, highlighting upwelling from the lower to the upper mantle by ridge suction force. The temperature anomalies are generally caused by the

latent heating around the phase boundaries of ~410 km and ~660 km, respectively. **c**, Effective viscosity field.

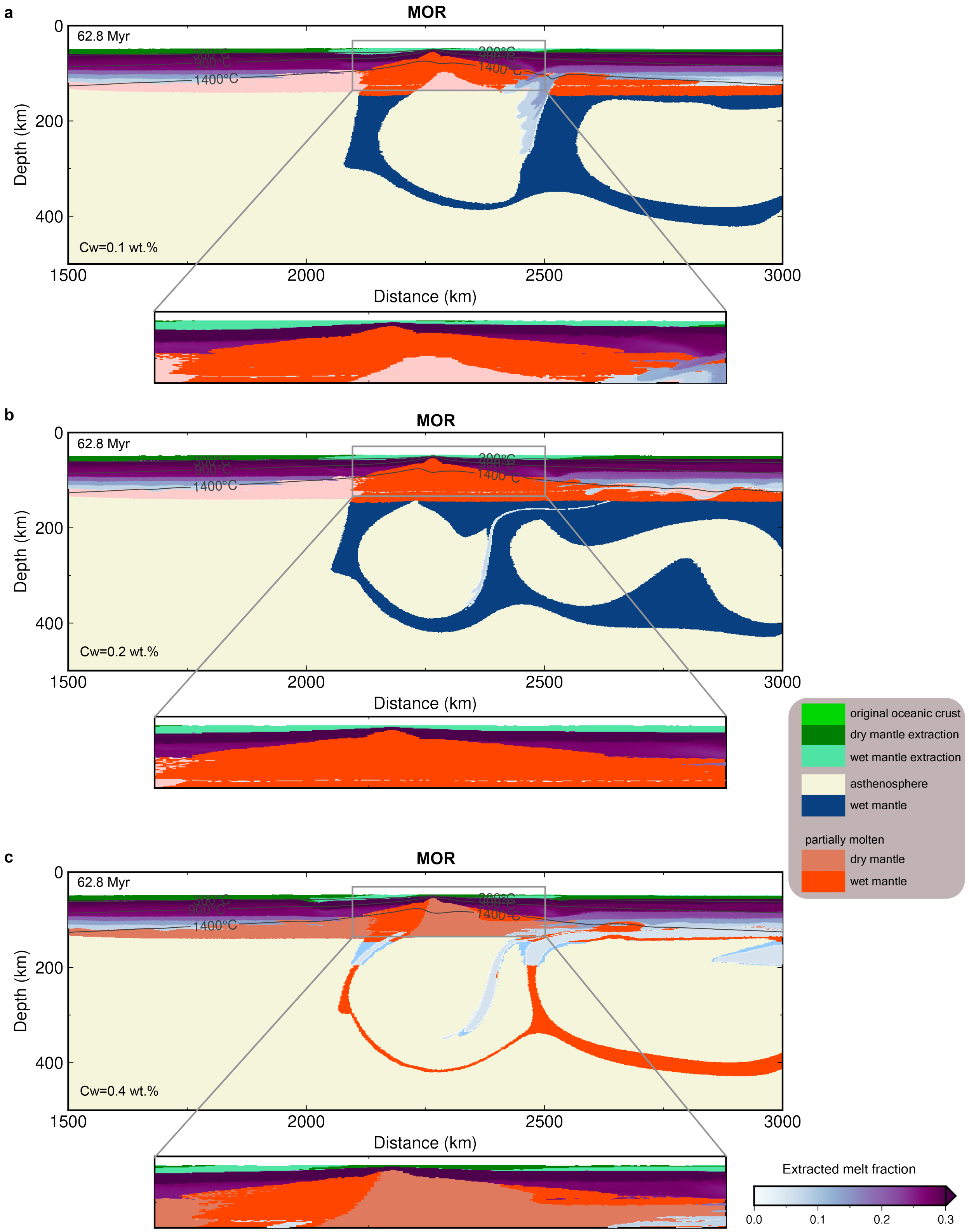


**Fig. S6 | Influence of the initial water content (wt.%) in the partially molten layer above the MTZ on the final mantle flow pattern.** The initial water content above the MTZ is **a**, 0.1 wt.%,

**b**, 0.2 wt.%, and **c**, 0.4 wt.%, respectively. For initial water contents below 0.3 wt.%, water release only metasomatizes the overlying mantle and drives solid-state upwelling without melting. Decompression melting solely occurs as the finger-like upwellings impinge on the oceanic lithosphere. As shown in the insets, the lithological structure beneath the ridge remains broadly organized into distinct horizontal layers. The convection cell between the two finger-like upwellings shrinks as water content increases. For a high-water content (0.4 wt.%), dehydration systematically triggers incipient melting as soon as the first Rayleigh instability develops from the hydrous layer above the MTZ. This process ultimately produces two upwellings of wet, partially molten mantle by the end of the simulation. As shown in the inset, the resulting mantle structure beneath the ridge is strongly asymmetrical, with wet mantle beneath the trailing flank and dry mantle under the leading flank. Other parameters are the same as the reference model if not described.

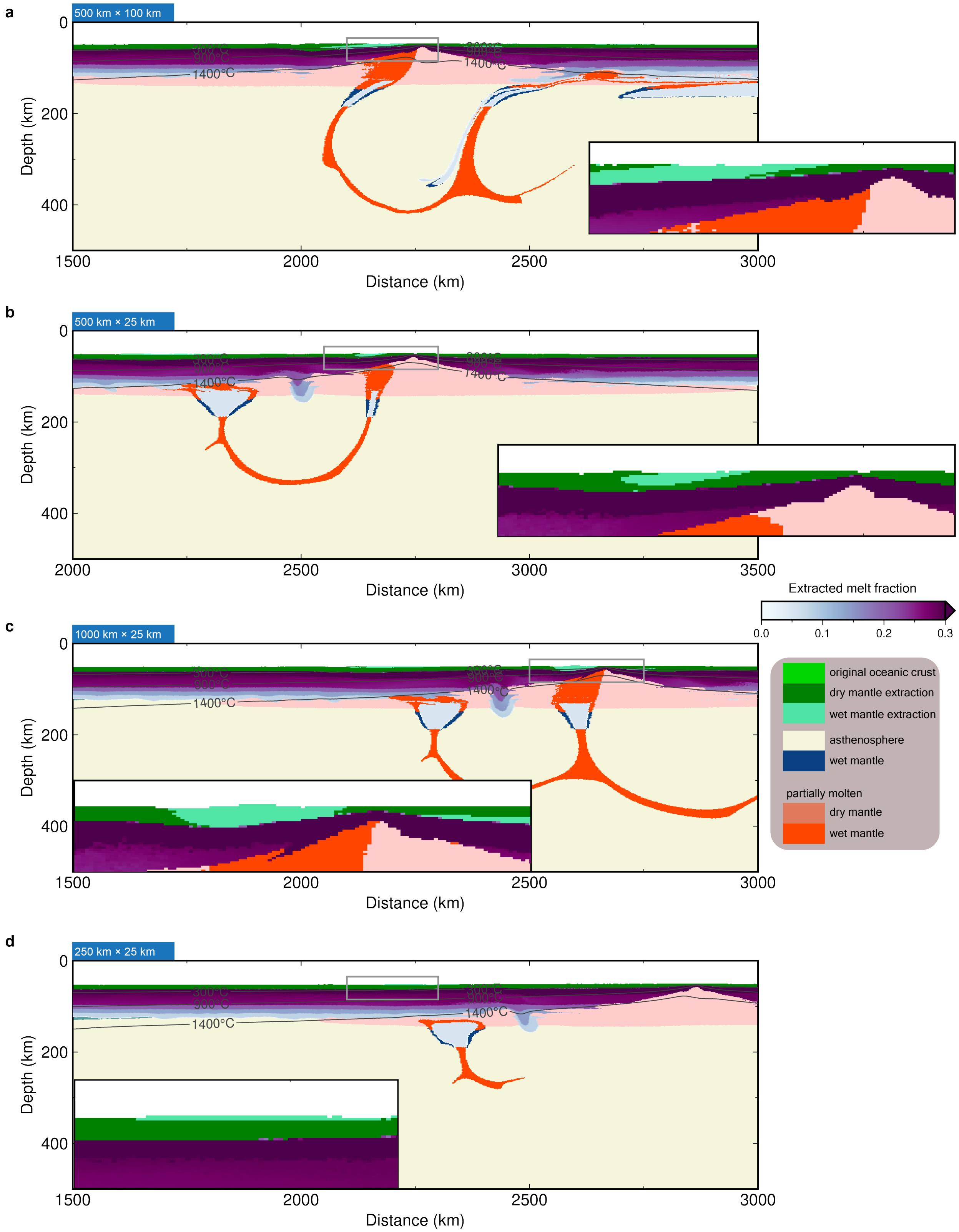


**Fig. S7 | Influence of the width and thickness of the partially molten layer above the MTZ on the final mantle flow pattern.** The wet zone is **a**, 500 km × 100 km, **b**, 500 km × 25 km, **c**, 1000

km × 25 km, and **d**, 250 km × 25 km, respectively. The insets are the zoom-in regions shown in the grey boxes. The initial water contents for the three models are 0.3 wt.% as the reference model in Fig. 2. Thinning the hydrous reservoir reduces the extent of the convective cell between the two finger-like upwellings and triggers Rayleigh instabilities at shallower depths. The secondary upwelling becomes strongly offset to the left beneath the stationary plate. The lateral asymmetry in the underlying mantle, with distinct wet and dry domains, is more marked for thicker and/or longer initial hydrous layers. A narrower (e.g., 500-km-wide) or thinner (e.g., 25-km-thick) hydrous reservoir produces less volcanism.

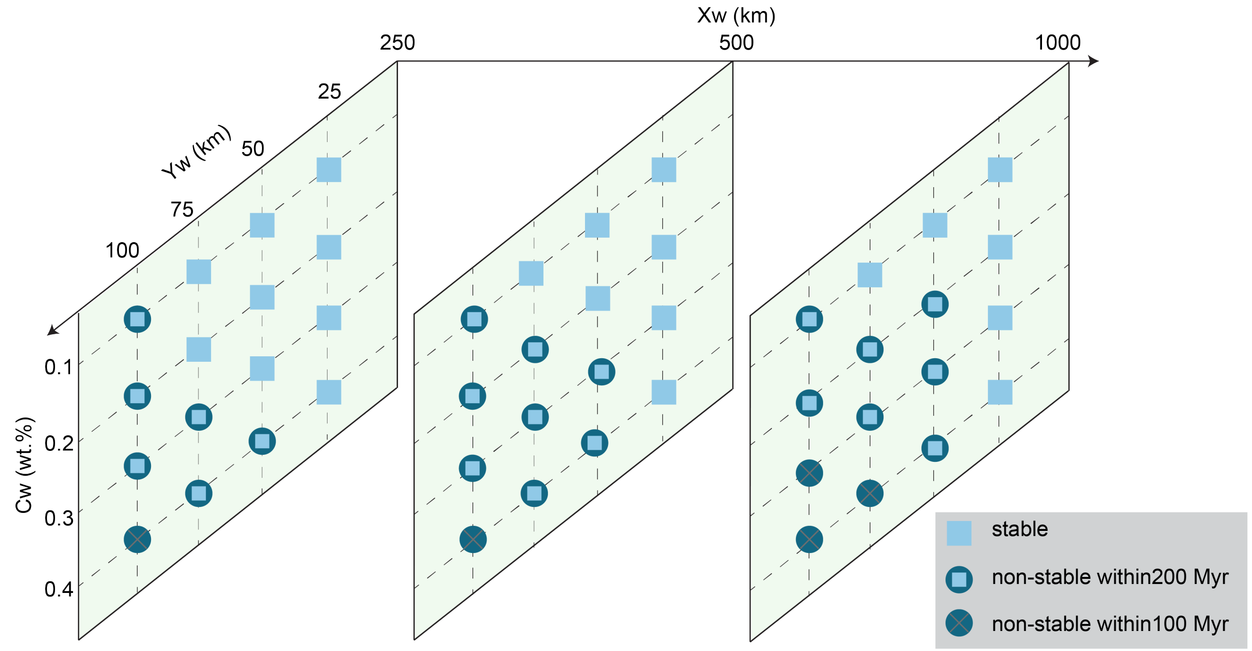


**Fig. S8 | Systematic parameter study of the stability of the hydrous layer above the MTZ as a function of its lateral extent (*Xw*), thickness (*Yw*), and water content (*Cw*) in a system without plate motion**. The hydrous reservoir remains stable when thin and weakly hydrated but becomes unstable within 100 Myr or 200 Myr if both the water content and thickness are relatively high.

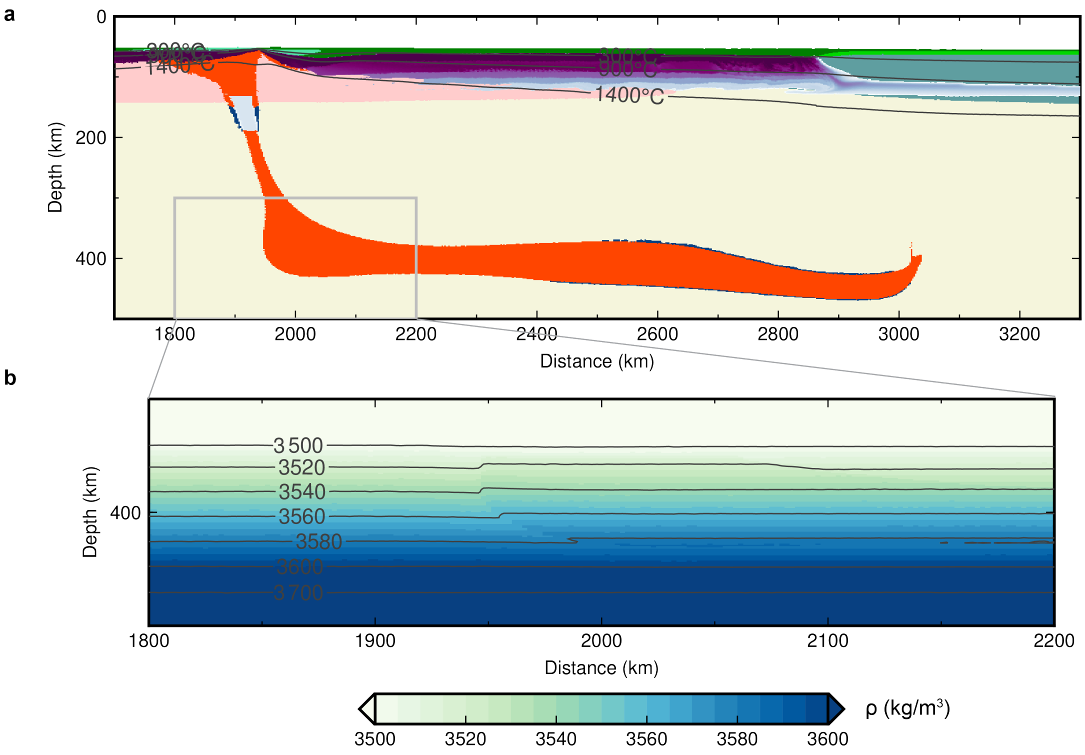


**Fig. S9 | Model result for a denser melt above 410-km discontinuity.** Assuming the melt density is 20% larger than the surrounding mantle based on the experimental studies, the other parameters are the same as that used in the reference model in Fig. 2. **a**, the composition field, and **b**, the zoom-in region for the density field. The contours for the density are labeled. Due to the low melt fractions, the density difference between the partially molten material and the surrounding mantle is minor.

**Table S1 | Physical properties of rocks used in this study.**

| Property | Symbol | Unit | Value |
|---|---|---|---|
| Gravity | $g$ | m $s^{-2}$ | 9.81 |
| Water content | $C_w$ | wt.% | - |
| Reference water content | $C_{w0}$ | wt.% | 0.001 |
| Melt fraction | $\phi$ | - | - |
| Melt-weakening factor | $\alpha$ | - | 28 |

| **Material** | **Flow law** |
|---|---|
| Oceanic crust | Wet quarzite, $A_d$=1.97×$10^{17}$ $Pa^n$ s, $n$=2.3, $E$=154 kJ $mol^{-1}$, $V$=8 J $mol^{-1}$ $MPa^{-1}$, $C$=1 MPa, $\mu$=0.05 |
| Weak zone mantle | $10^{18}$ Pa s |
| Lithospheric/asthenospheric mantle | Dry olivine from ref[9], $C$=20 MPa, $\mu$=0.6<br>Diffusion creep: $A_d$=8.7×$10^{15}$ Pa s, $G$=80 GPa, $E$=300 kJ $mol^{-1}$, $V$*, $r$=0.8, $b$= 0.5 nm, $d$=1 mm, $m$=2.5<br>Dislocation creep: $A_d$=3.5×$10^{22}$ $Pa^n$ s, $n$=3.5, $G$=80 GPa, $E$=540 kJ $mol^{-1}$, $V$=18 J $mol^{-1}$ $MPa^{-1}$, $r$=1.2 |

*Given the uncertainty in the activation volume of diffusion creep for dry olivine and considering that diffusion creep likely dominates deformation in the lower mantle, the activation volume $V$ is assumed to decrease linearly from 5 J $mol^{-1}$ $MPa^{-1}$ to 3.75 J $mol^{-1}$ $MPa^{-1}$.

**Supplementary References**


1. Asimow PD, Langmuir, CH. The importance of water to oceanic mantle melting regimes. *Nature* **421**, 815-820 (2003).
2. Asimow PD, Dixon J, Langmuir C. A hydrous melting and fractionation model for mid-ocean ridge basalts: Application to the Mid-Atlantic Ridge near the Azores. *Geochemistry, Geophysics, Geosystems* **5**, (2004).
3. Herzberg C, Gazel E. Petrological evidence for secular cooling in mantle plumes. *Nature* **458**, 619 (2009).
4. Beier C, Haase KM, Turner SP. Conditions of melting beneath the Azores. *Lithos* **144**, 1-11 (2012).
5. Dixon JE, et al. Light stable isotopic compositions of enriched mantle sources: Resolving the dehydration paradox. *Geochemistry, Geophysics, Geosystems* **18**, 3801-3839 (2017).
6. Métrich N, Zanon V, Créon L, Hildenbrand A, Moreira M, Marques FO. Is the 'Azores hotspot'a wetspot? Insights from the geochemistry of fluid and melt inclusions in olivine of Pico basalts. *J Petrol* **55**, 377-393 (2014).
7. Hosseini K, Matthews KJ, Sigloch K, Shephard GE, Domeier M, Tsekhmistrenko M. SubMachine: Web-based tools for exploring seismic tomography and other models of Earth's deep interior. *Geochemistry, Geophysics, Geosystems* **19**, 1464-1483 (2018).
8. Zhou J, Liu J, Xia Q, Su C, Kuritani T, Hanski E. A Machine Learning Based-Approach to Predict the Water Content of Mid-Ocean Ridge Basalts. *Geochemistry, Geophysics, Geosystems* **24**, e2023GC010984 (2023).
9. Karato S-i, Wu P. Rheology of the upper mantle: A synthesis. *Science* **260**, 771-778 (1993).
