# Supplementary material for: Subduction legacies in the mantle transition zone modulate intraplate oceanic volcanism
Source: Nat Commun. 2026 May 18;17:6566. doi: 10.1038/s41467-026-73403-7 (PMC13381961; doi:10.1038/s41467-026-73403-7)
Supplement: Supplementary file 2 — Description of Additional Supplementary Files [file 41467_2026_73403_MOESM2_ESM.pdf]

Description of Additional Supplementary Files:

Supplementary Video 1: The model evolution of the composition field for the reference model.
