# Supplementary material for: Subduction legacies in the mantle transition zone modulate intraplate oceanic volcanism
Source: Nat Commun. 2026 May 18;17:6566. doi: 10.1038/s41467-026-73403-7 (PMC13381961; doi:10.1038/s41467-026-73403-7)
Supplement: Supplementary file 4 — Transparent Peer Review file [file 41467_2026_73403_MOESM4_ESM.pdf]

nature portfolio

Peer Review File

Subduction legacies in the mantle transition zone modulate intraplate oceanic volcanism

Corresponding Author: Dr Jianfeng Yang

This file contains all reviewer reports in order by version, followed by all author rebuttals in order by version.

Version 0:

Reviewer comments:

Reviewer #1

(Remarks to the Author)
The authors challenge the generally accepted notion of a plume head for oceanic plateaus. It is unclear what the authors mean by "…multidisciplinary evidence has yet to fully support the plume head hypothesis". What evidence are missing? What is the problem with the current hypotheses? The paper would benefit from defining what the authors mean by 'hot plume' throughout the text. I suspect they mean a pure thermal anomaly that is anchored to the core-mantle boundary.

Overal, the manuscript is based on a previously published geodynamic model from the first two authors. As described, the paper really has little to do with the Azores; the model and content of the paper can be applied anywhere where a ridge is moving over a proposed wet MTZ. This then creates the question: if a migrating ridge creates suction above a wet MTZ, why are we not seeing AZORES-like features everywhere along ridges? What is special about the Azores? Perhaps a hot plume anomaly is indeed required, contrary to what the authors propose. Or, the MTZ is not as wet as the authors model it, and it is only happening in the Azores.
I cannot really comment the modeling aspect as it is not in my field. But as a geochemist, and from the cartoons in Figure 2 and the text, I see no reason why this would only happen in the Azores (the Farralon subduction is really not a good explanation as the size of that slab is much wider than the localized melting in the Azores). This needs to be addressed.

Reference #19 is incomplete.

Line 71. hydrated layer of what? Is this layer a mantle peridotite of ambient composition, with excess water? Or something else? Please clarify in the text.

72-73. I am sure this has been presented in other papers, but it is unclear how this model works in this context, and needs a better description. A hydrated mantle may melt if it is disturbed with respect to its solidus. It will create a melt that will escape. How will this "…degree of hydration trigger melting in the overlying mantle"? Is water being transferred from the hydrated to the dry mantle? How? Do the extracted melts reequilibrate with the overlying drier mantle? Or is it just the "wet" portion on top of the MTZ that melts? There are pieces of the puzzle missing here, therefore I do not understand how valid the proposed model is.

117: Why would you change the Mg content? What would control that? And in the next sentence, the part : "…making its effect negligible at ambient mantle temperature.." is not understood. Please explain.

124: Higher water contents in what? In the basalts of the plateau?

128: If the local adiabat is hotter by 100 C or more, as implied here as an alternative, what is the effect of a hotter (and presumably deeper) plume that penetrates the proposed wet MTZ? For the paper to fully address the proposed wet MTZ source, it must account for the effect of a deeper hot plume penetrating through the MTZ. If an upwelling deeper plume that penetrates and interacts with the proposed wet MTZ produces significantly different outcomes inconsistent with observations, then that would substantially support the authors' point. At the very least, this needs to be discussed in the supplement.

137-141. As written, the text takes a leap of faith. First, it mentions the evidence for a thin layer of low seismic anomalies

above the MTZ. That is fine. But the next sentence, "…This hydrous melting enables widespread intraplate volcanism…", presents as evidence the thing the authors are trying to prove (a link between deep wet MTZ and shallow mantle melting). The authors have not sufficiently explained the gap between a wet MTZ top at ~ 410 km and melting at 120 km. There is nearly 300 km of mantle in between, where, somehow, the proposed hydrous melt at the top of the MTZ is creating a new source that will then melt at shallower depths. This part needs more explanation.

Text in 152-155 is just speculative.

163. Which plateaus?

165-167. This speculative sentence basically says that everything is possible. Not very satisfying.

170-171: "…mantle rehydration via MTZ dehydration melting…". I think this is a key mechanism that requires further explanation.

178: What alternative does it offer? It still requires a plume (high 3/4He or primordial He as the authors say) + recycled materials. That has always been the explanation. Please clarify.

Reviewer #2

(Remarks to the Author)
The manuscript entitled "Subduction legacies in the mantle transition zone modulate intraplate oceanic volcanism" by Yang and co-authors deals with the problem of the origins of oceanic plateaus – and in particular the Azores Plateau – and ocean island volcanism in what concerns their origin by mantle plumes, which the authors argue could also be explained by migration of plates over hydrous sections of the Mantle Transition Zone, which in turn they argue is the legacy of previously subducted slabs. The paper thus deals with a relevant, timely and hot topic in geodynamics, and as such brings a refreshing and different perspective to the debate. In support of their scientific discourse, the authors use model results derived from the widely used I2VIS, two-dimensional (2D) thermo-mechanical geodynamic numerical code developed by Taras Gerya and David Yuen, relating their observations with other independent observations from the literature, namely on the water content and geochemical signature of melts. Personally, I enjoyed reading the manuscript and was encouraged by the novel perspective. If the authors are right, what they propose is far-reaching and with key consequences for what we know about the formation of oceanic plateaus, island volcanism and mantle melting. I think however, this study is not yet at the state of maturity to offer a solid contribution to the debate despite its excellent potential, particularly taking into account the high standards of Nature Communications. I therefore recommend major revisions and leave here a few aspects as food for thoughts in what I consider to be weaknesses or points for improvement in the paper.

[1] first and foremost, I find the way the model results are displayed in figure 2 – which is absolutely central to the paper – really difficult to read, making it hard to see how what is observed in the figure may or may not support the far-reaching statements found in the text. This is partially due to the choice of colours, lack of velocity field vectors in the entirety of the model panels (limiting the reader's capacity to understand and interpret the data using the main figures of the paper), difficulty in reading the isothermal lines and labels, and also the differentiation of the different fields displayed in the figure, which I find really confusing (see my comments on the manuscript). For the manuscript to be published in Nature Communications I suggest the authors overall the way they display the model and significantly improve the readability of the figure, which should be self-explanatory and at this stage (perhaps is just me) requires quite a lot time to digest and understand.

[2] second it is not clear to the reader – just on the basis of this figure – how exactly the model supports the formation of a plateau such as the Azores plateau - you need to find a more effective way to link the model runs (perhaps through a more effective labelling) to make this link more evident for the less specialist readership. In this respect the video seems to be a bit more evident. I acknowledge that I am not geodynamic modeller but more so the model should be better comprehensible (or more readable) by a less niche reader such as me.

[3] third, if I understood the model correctly, the model implies hydrous melting and hydrous volcanism at the MOR but only for a period (presumably when the plateau is generated) – where is the evidence for that? I.e. where is the evidence that the lavas that formed the Azores plateau are particularly rich in volatiles and as such reflect an hydrous mantle source? And notwithstanding the fact that many Azores Island lavas (but not the plateau lavas, from my knowledge) reflect a volatile-rich source, there is also evidence from noble gas geochemistry suggesting a thermal (or thermochemical) mantle plume (e.g. Madureira et al, 2005; Madureira et al., 2014; Moreira et al 2018)... how do you conciliate these different pieces of evidence?

[4] more broadly – and more importantly due to the implications the arguments bring - your model basically implies that most intra-plate volcanism can me potentially explained by melting taking place in hydrous zones of the MTZ... at least this is what the title and some statements imply. How do you conciliate this with evidence from melt geochemistry and seismic tomography at other intra-plate localities that strongly suggest the presence of mantle plumes deeply anchored in the CMB and for melting to reflect primitive rather than recycled sources (e.g Iceland, Hawaii, Canaries/Cape Verde)? I particularly feel this part of the debate in the paper is not fully supported by evidence from the literature..

[5] what the authors describe as "upwelling" from the MTZ are effectively – or geometrically – what others describe as plumes, even if these are only anchored at the MTZ rather than the CMB... Therefore I think the use of the terms here is

perhaps not entirely appropriate although I partially understand why the authors avoid using the term "plumes" to describe the (presumably columnar) upwelling they observe in the their models.

I leave these comments here as some food for thought, hopefully they all will help improve a manuscript that despite being (in my view) not yet ready for publication, when it is ready and improved may stir the debate on the formation of oceanic plateaus and ocean island volcanism, providing a provocative alternative explanation to these features.

Yours truly,

Ricardo Ramalho

Reviewer #3

(Remarks to the Author)
In this work, Yang and co-workers challenge the classic mantle plume origin for oceanic plateaus by proposing an alternative mechanism, namely that a hydrated mantle transition zone (MTZ) can create localized upwellings of increased melt, which leads to long-term volcanism and anomalous crustal thickness.
Their results seem to validate this mechanism, where the formation of localized upwelling derived due to adiabatic decompression in the vicinity of the mid-ocean ridge can be seen. According to the authors, these results are similar to what can be observed in the Azores Plateau.
The manuscript was quite interesting to read, but I do have some questions and comments regarding their work:
1. While I agree with the authors' assessment that the classic mantle plume model (i.e., a deep-rooted thermal anomaly, stemming from the core-mantle boundary) does not explain the observed anomalies at the Azores, I do believe that what they model and show is, for all effects and purposes, a plume. It is described by the authors as a Rayleigh-Taylor instability which upwells to the surface (ln. 75-77), which is exactly what a thermal plume would do. In fact, this is clear by observing Fig. 2, where two clear mushroom-like anomalies ascend through the upper mantle. In my view, this work contributes towards expanding our understanding of mantle plumes, showing a different way of forming them at lower depths along the MTZ and I believe the authors should lean into this aspect. If they disagree, I would at least like to know their opinion.
2. On the topic of looking at figures, I don't entirely follow the authors' reasoning with Figure 1. The authors state that there are "elevated H2O/Ce ratios of both OIBs and MORBs from the Azores compared to background MORB ratios to the south" (ln. 42-43), however there doesn't seem to be a significant difference between the values for the Azores and the rest of the data points. In fact, they seem to plot very much along the trend shown. Given that the increased water content seems to induce upwellings, following this reasoning I would expect significant intraplate volcanic activity in the mid Pacific. Could the authors comment on this? Also on this figure, there are no red squares as indicated by the caption, probably a typo.
3. Continuing to Figure 2, I find the colour legends applied to be unclear. What applies to what? It appears to be a superposition between the composition colours and the melt fractions which makes it unclear to me. This makes it quite hard to observe the crustal thicknesses described in the text. This could be solved by adding a scale to the insets on the right.
4. Still in Figure 2, on 2c) and onwards, I see that there is a region of zero melt wet mantle within the plumes, between ca. 100 and 180 km deep. What is the reason for this? The linear aspect of it suggests that it might be a phase transition or pressure effect, but I couldn't find an explanation for it in the manuscript.
5. Later stages of the authors' model show an upwelling within the mid-ocean ridge (MOR) which, in my view, would have to imply result in water-rich basalts being formed at the MOR itself. I saw some references in the ms to water-rich basalts in cold spots of the Mid-Atlantic Ridge (ref. 40 I believe?), but this is significantly further south. Is there evidence for hydrated basalts at the MOR in the Azores? If not, the authors should discuss this somewhere, perhaps in a model limitations section.
6. While the authors show the thermal fields for the last stages of the model in Fig. S3, I'd like to see it for the remaining stages, particularly for 53 Myr stage. By the final stage there is a near total recuperation of the initial temperature profile (plus or minus a few tens of degree) but, from what I can see in Fig. 2c-d, the 900C isotherm here is much lower than on the other figures so I'd like to understand this discrepancy.

I believe this paper to be of great importance to the field of geodynamics; however, the additional clarifications suggested will increase its impact and clarity for the potential reader. I am confident that addressing these issues will make manuscript will be a worthy contribution to this journal.
The authors should feel free to contact me if they have any questions regarding my review.

Best regards,
Jaime Almeida

Version 1:

Reviewer comments:

Reviewer #1

(Remarks to the Author)
I commend the authors for their diligent work in the revision. I find that the paper now is more "honest", meaning that it explores more caveats and addresses some inconsistencies or things that were unclear. The paper provides evidence for an additional mechanism to understand intraoceanic excess volcanism, particularly one that is assisted by a migrating ridge.

The one thing that is quite pertinent in the Azores plateau, and my apologies for not thinking about in the first review, is the triple junction of the Atlantic ridge and the Terceira Rift. Work by Georgen (e.g. Georgen EPSL 2008, and some other work), have shown that the triple junction may result in excess melting as a result of the three plate spreading, and therefore excess crust production. Understandably, a 3D flow model may be significantly different than the model in this paper. Nonetheless, as the manuscript describes the excess magma at the Azores triple junction, it should also discuss that other models have attributed excess crustal production to the Triple junction configuration, albeit assisted by a nearby "hot plume". Can the authors speculate how a triple junction mantle flow affect their model calculations and if some of the excess "crust" is due to the mantle flow assisted by the Triple junction, what / how could it affect their model input (perhaps the amount of water in the source, if water drives degree of melting)?

Reviewer #2

(Remarks to the Author)
I am happy with the authors' response and changes in the manuscript – I believe they address all reviewers' concerns in a satisfactory manner, despite some still existing weaknesses (lack of evidence for wider spread hydrated melts in the plateau and islands – so far evidence is restricted to OIB lavas from Pico and offshore. The manuscript is solid enough to stir the debate and provide an alternative model for the formation of the Azores and the most excessive comments were toned down or modified to make them clearer. I am therefore happy for the manuscript to be published as it is.

Reviewer #3

(Remarks to the Author)
The authors have adequately addressed all my comments and questions. The clarity of both the text and figures of the manuscript have significantly increased during the review.

It is my opinion that the manuscript is now ready for publication.

Best regards,
Jaime Almeida

Open Access This Peer Review File is licensed under a Creative Commons Attribution 4.0 International License, which permits use, sharing, adaptation, distribution and reproduction in any medium or format, as long as you give appropriate credit to the original author(s) and the source, provide a link to the Creative Commons license, and indicate if changes were made.
In cases where reviewers are anonymous, credit should be given to 'Anonymous Referee' and the source.
The images or other third party material in this Peer Review File are included in the article's Creative Commons license, unless indicated otherwise in a credit line to the material. If material is not included in the article's Creative Commons license and your intended use is not permitted by statutory regulation or exceeds the permitted use, you will need to obtain permission directly from the copyright holder.
To view a copy of this license, visit https://creativecommons.org/licenses/by/4.0/

Reviewers' comments:

Reviewer #1 (Remarks to the Author):

The authors challenge the generally accepted notion of a plume head for oceanic plateaus. It is unclear what the authors mean by "…multidisciplinary evidence has yet to fully support the plume head hypothesis". What evidence are missing? What is the problem with the current hypotheses?

R: We thank the reviewer for this important question. By stating that "multidisciplinary evidence has yet to fully support the plume head hypothesis," we refer to the fact that several key predictions of the classical thermal plume model are not consistently observed in drilling and geophysical studies of major oceanic plateaus.

For example, scientific drilling at the Ontong Java Plateau, Shatsky Rise, and Kerguelen Plateau (ODP Legs 183, 192; IODP Expedition 324) has revealed complexities difficult to reconcile with a simple plume head, such as the lack of requisite subaerial exposure, evidence for cooler-than-expected melting temperatures, and the clear tectonic association with spreading ridges and triple junctions (e.g., Korenaga, 2005; Sager et al., 2016; Neal et al., 2019). A striking observation is that most plateaus, including the Azores, Ontong Java Nui, and Rio Grande Rise, formed in near-ridge settings, implying a fundamental interaction with plate-driven flow that our model tried to address.

In the revised manuscript, we have substantially expanded the "Implications for the thick crust in the Azores Plateau" section (Lines 116-151) to synthesize the geological, geochemical, and geodynamic context that challenges a simple thermal plume origin for the Azores. This now includes a discussion of: (1) the tectonic context and ridge interaction in the Azores; (2) thermal and compositional constraints (the geochemical evidence for a water-rich, recycled mantle source rather than an ultra-hot thermal anomaly); (3) seismic structure (low-velocity anomalies constrained in the upper mantle rather than the deep-rooted plume). We then demonstrate how our model of hydrous MTZ upwelling provides a coherent alternative (also in the next section for discussion).

The paper would benefit from defining what the authors mean by 'hot plume' throughout the text. I suspect they mean a pure thermal anomaly that is anchored to the core-mantle boundary.

R: We thank the reviewer for this helpful suggestion. The reviewer is correct: throughout the manuscript, we use the term "hot plume" or "classical mantle plume" to refer to a deep-rooted, thermally buoyant upwelling originating from the core-mantle boundary (CMB), characterized by a significant potential temperature anomaly (typically >1400–1500°C) relative to ambient mantle.

To avoid ambiguity and align with the reviewer's interpretation, we have now added a clear definition at the beginning of the manuscript (Lines 31-33). Specifically, we define the **Deep**

thermal plumes: Buoyant upwellings originating from thermal boundary layers (e.g., CMB) with excess temperatures of 200–300°C.

In contrast, the mechanism proposed in this study: upwellings triggered by buoyancy from hydration and dehydration melting in the MTZ, which operate at ambient or only modestly elevated mantle temperatures (1350–1400°C). Thus, we use "hydrous upwelling" throughout the MS.

We believe this clarification addresses the reviewer's concern and helps frame our model as an alternative driver of upwelling, rather than a rejection of the existence of upwellings themselves, as we emphasized in Lines 210-218.

"We emphasize that hydrous MTZ upwelling is not proposed as a replacement for mantle plumes in all intraplate settings…"

In the abstract we also now state:

"...providing an alternative to the classical stationary mantle plume model…"

Overal, the manuscript is based on a previously published geodynamic model from the first two authors. As described, the paper really has little to do with the Azores; the model and content of the paper can be applied anywhere where a ridge is moving over a proposed wet MTZ. This then creates the question: if a migrating ridge creates suction above a wet MTZ, why are we not seeing AZORES-like features everywhere along ridges? What is special about the Azores?

R: We thank the reviewer for this insightful question. Compared to the subduction models by Yang and Faccenda, 2020 (YF2020), these are very different geodynamic models that were specifically designed to study intraoceanic settings, including the Azores. In YF2020 we purposely defined a homogeneously wet MTZ to understand where potentially intraplate volcanism could form upon subduction-induced upwellings (the forces are originating from the subduction zones, not from the ridge. Therefore, they are completely different tectonic settings), but obviously that was an upper bound (although not unrealistic as we only imposed 0.3 wt% or less water) causing lots of intraplate volcanism and we are aware that the situation is likely more heterogeneous.

In this study, instead, we define more realistic and laterally limited wet zones, with a geometry that is consistent with seismic observations of low-velocity anomalies atop the 410 km (e.g., Tauzin et al., 2010). In addition, in YF2020, the upwellings are triggered by subduction, while here by the suction generated by the migrating spreading ridge. This is the true novel aspect of the model that, although consistent with all the other numerical solutions, was overlooked in previous studies.

The Azores region is of particular interest for our model because its magmatism has long been shown to reflect excess water rather than elevated mantle temperature (e.g., Bonatti, 1990; Kingsley and Schilling, 1995; Asimow et al., 2004; Asimow and Langmuir, 2003; Herzberg and Asimow, 2008; Gazel and Herzberg, 2008; Metrich et al., 2014), making it a canonical example of a "wetspot" (Bonatti, 1990) and an ideal test case for our model. The Azores plateau is among

the rare oceanic plateaus for which an extensive dataset of water contents exists, including measurements from on-axis MORB (Michael, 1995; Dixon et al., 2002, 2017) as well as OIB from Pico Island (Métrich et al., 2014). In contrast, water-content data are extremely sparse for most oceanic plateaus, whether young or ancient (Portnyagin et al., 2008; Koleszar et al., 2009; Michael, 1999; Roberge et al., 2004; Timm et al., 2011), severely limiting quantitative tests of our model. In addition, the youth of the plateau provides an exceptional natural laboratory for quantifying how ridge migration relative to a hotspot over time influences the processes that lead to plateau formation. Quantifying ridge-hotspot interactions is generally difficult because the original tectonic setting of most plateaus is poorly constrained. Many formed during the Cretaceous Quiet Zone and therefore lack ridge-parallel magnetic anomalies that would otherwise record ridge positions.

The reviewer is correct that our model could, in principle, apply to any ridge migrating over a **hydrated** MTZ. However, the MTZ is not uniformly hydrated. As shown in Fig. 1a, seismic estimates of water content in the MTZ reveal significant lateral heterogeneity (although this interpretation of the water content from seismic imaging studies is also debated). Most regions beneath MORs appear to have lower water contents, which would not generate sufficient buoyancy or melting to produce a thick plateau (see Fig. 3 & S4, which show crustal thickness sensitivity to water content).

In the revised manuscript, we have clarified these points in Lines (160-165, 210-218) that address the global applicability and limitations of our model. We now explicitly state that the Azores represent a "typical example" where the necessary conditions coincide, rather than claiming that all plateaus form this way.

Perhaps a hot plume anomaly is indeed required, contrary to what the authors propose. Or, the MTZ is not as wet as the authors model it, and it is only happening in the Azores.

R: We thank the reviewer for raising these two fundamental questions.

1) We may not require a thermal anomaly. Multiple independent petrological studies indicate that the mantle beneath the Azores does not exhibit the extreme potential temperatures (ΔTp >200°C) characteristic of deep thermal plumes. Anhydrous melting models applied to Azores lavas yield mantle potential temperatures (Tp) that overlap substantially with ambient mantle values (1342–1461°C; Gazel and Herzberg, 2008). Even the most generous estimates of thermal excess (ΔTp) are modest (≤75°C; Beier et al., 2012; Asimow and Langmuir, 2003). When water is accounted for, the required Tp decreases further (Asimow et al., 2004), making a purely thermal plume incompatible with the observations. Instead, the low Tp estimates, combined with elevated volatile contents, point toward a water-fluxed melting regime rather than a hot, dry upwelling.

2) We argue that the Azores are a good example of a broader phenomenon. The water content (and also H_2O/Ce ratios; Fig. 1b & Fig. S2) in Azores MORB and OIB (Métrich et al., 2014) is high, but they are not unique. Similar spikes in H_2O/Ce occur elsewhere, such as at the Equatorial Atlantic Discordance (Ligi et al., 2005), where independent seismic imaging reveals MTZ thinning consistent with upwelling (Agius et al., 2021). These observations suggest that localized wet domains in the MTZ are more common than previously recognized, but their detection requires

high-resolution geochemical and geophysical data that are not available for most oceanic regions. The Azores thus serve as a good example, rather than an isolated exception.

In the revised manuscript, we have added a concise summary of this petrological evidence in Lines 140-151 and clarified that the Azores represent the best-studied, but likely not the only, manifestation of MTZ-derived hydrous upwelling (Lines 181-184).

I cannot really comment the modeling aspect as it is not in my field. But as a geochemist, and from the cartoons in Figure 2 and the text, I see no reason why this would only happen in the Azores (the Farralon subduction is really not a good explanation as the size of that slab is much wider than the localized melting in the Azores). This needs to be addressed.

R: We agree with the reviewer that the Farallon slab, as a subduction system, was vast. However, MTZ hydration is not expected to be uniform across the entire slab footprint. Dehydration of the slab during subduction is heterogeneous, controlled by variations in slab temperature, composition, and structure (van Keken et al., 2011), as well as by the degree of oceanic plate hydration, which depends on several processes occurring at the surface (Faccenda, 2014). This produces a heterogeneous distribution of hydrated domains within the MTZ, rather than a single, uniformly wet layer. The localized nature of the Azores volcanism (and similar features like Bermuda) reflects the exploitation of one such localized, highly hydrated "sweet spot" by the migrating ridge. The broad Farallon slab provides the source of water, but tectonic and dynamic processes determine where that water is concentrated and subsequently mobilized.

This heterogeneity is consistent with geochemical observations: the Azores lavas do not sample a uniform "Farallon" signature, but instead show isotopic variations between islands that suggest sampling of distinct, small-scale heterogeneities within a broader recycled domain. Our model captures this by imposing a laterally restricted wet zone (1000 km wide, 75 km thick), which is consistent with seismic images of low-velocity layers atop the 410-km discontinuity that are also laterally discontinuous.

In the revised manuscript, we have added a brief discussion (Lines 187-193) clarifying that the Farallon slab provides the water reservoir, but that hydration of the MTZ is heterogeneous, and ridge suction mobilizes only the most fertile, localized domains.

Reference #19 is incomplete.

R: This reference has been corrected.

Line 71. hydrated layer of what? Is this layer a mantle peridotite of ambient composition, with excess water? Or something else? Please clarify in the text.

R: Yes, the reviewer is correct: the hydrated layer in our model consists of mantle peridotite of ambient composition, but with excess water stored in nominally anhydrous minerals (NAMs).

Specifically, we impose a water content of 0.3 wt.% H_2O within this layer (but also test different amounts of water contents), which is substantially higher than the background upper mantle (typically ≤0.1 wt.% H_2O) but consistent with experimental constraints on water storage capacity in MTZ minerals (wadsleyite and ringwoodite can accommodate up to 1–3 wt.% H_2O; Kohlstedt et al., 1996). This excess water lowers the solidus and promotes melting when the material upwells.

We have now clarified this in the text (Line 80) as follows:

"...imposes a 1000-km-wide, 75-km-thick layer of mantle peridotite with excess water (0.3 wt.% H_2O stored in NAMs) atop the MTZ..."

72-73. I am sure this has been presented in other papers, but it is unclear how this model works in this context, and needs a better description. A hydrated mantle may melt if it is disturbed with respect to its solidus. It will create a melt that will escape. How will this "…degree of hydration trigger melting in the overlying mantle"? Is water being transferred from the hydrated to the dry mantle? How? Do the extracted melts reequilibrate with the overlying drier mantle? Or is it just the "wet" portion on top of the MTZ that melts? There are pieces of the puzzle missing here, therefore I do not understand how valid the proposed model is.

R: We apologize for the confusion and have revised the text to provide a clearer explanation of the physical process. We address each of the reviewer's specific questions below.

1) The reviewer is correct that it is the wet portion itself that melts. There is no transfer of water from the wet, partially molten layer to the overlying dry mantle in our models (i.e., water is preferentially partitioned into the melt). The phrase "trigger melting in the overlying mantle" in the original text was imprecise. We have removed it and now describe the process more accurately as decompression melting of the hydrous upwelling as it rises.

2) When the hydrated peridotite resides at the base of the upper mantle (410 km depth), the melt fraction is extremely low (≤1–2% for 0.1–0.3 wt.% H_2O). At such low melt fractions, the melt is interconnected but cannot escape the solid matrix due to high viscosity and low permeability. The partially molten material thus remains a two-phase mixture that is buoyant relative to the dry mantle.

3) Melting is triggered by either dehydration melting or decompression melting. If the initial water content is relatively high, it likely results in dehydration melting (as shown in our reference model for 0.3 wt.% H_2O). As the hydrated or partially molten material rises, the melt fraction increases. Only when the partially molten rock fits a certain criterion, melt extraction is allowed (depending on the melt fraction, melt and mantle viscosity, and so on, which is described in the Methods). This extracted melt is assumed to be instantaneously emplaced at the surface to form new crust.

In the revised manuscript, we have added a concise explanation of this four-step process, labeled in Fig. 2 (and described in the Methods section, Lines 500-508), to ensure readers understand the physical chain of events from initial hydration to final melt extraction.

1. initial partially molten wet mantle

2. hydrous upwelling

3. ridge-induced suction and enhanced melting

4. thick crust formation

117: Why would you change the Mg content? What would control that? And in the next sentence, the part : "…making its effect negligible at ambient mantle temperature.." is not understood. Please explain.

R: We apologize for the confusion; we are not changing the Mg content in our models. The text at Line 117 discussed the broader geophysical literature on factors influencing the depth of the 410-km discontinuity, not our model parameters.

The mention of Mg content refers to experimental and seismic studies showing that variations in mantle composition (specifically the Mg# of olivine) can shift the pressure of the olivine-to-wadsleyite phase transition, thereby affecting the apparent depth of the 410-km discontinuity (e.g., Smyth and Jacobsen, 2006). We introduced this to acknowledge that interpreting MTZ topography is complex and that multiple factors (temperature, composition, and water) can trade off with one another. We do not vary Mg content in our models.

On the phrase "making its effect negligible": This sentence was poorly phrased. We meant that the effect of water on the depth of the 410-km discontinuity becomes negligible at ambient mantle temperatures because the solubility of water in olivine and wadsleyite decreases with increasing temperature (Frost, 2008). In other words, a warm mantle cannot retain significant water, so any hydration effect on discontinuity depth would be minimal unless the mantle is relatively cool. This is why we consider our ambient temperature assumption (1350°C) reasonable for testing hydrous models.

We have now rewritten these sentences (Lines 134-139) to make it clear that we are discussing literature constraints, not model inputs, and to explain the temperature-solubility relationship more clearly.

124: Higher water contents in what? In the basalts of the plateau?

R: Yes, the reviewer's understanding is correct. The comparison refers to water contents measured in basalts from the Azores Plateau (both MORB and OIB) versus those from our models. Specifically, Asimow & Langmuir (2003) and Métrich et al. (2014) estimate mantle source water contents of 0.05–0.09 wt.% H_2O for Azores magmas based on petrological modeling, whereas our

geodynamic simulations require slightly higher source water contents (0.1–0.4 wt.% H_2O) to reproduce the observed crustal thickness.

To avoid ambiguity, we have revised the sentence at Line 153 to explicitly state:

"Our models reproduce the thick crust observed at the Azores (e.g., 10.4±2.1 km for 0.3 wt.% H_2O at 1350ºC), albeit with higher mantle source water contents than those inferred from shallow wet melting models (0.05–0.09 wt.% H_2O in the source)."

128: If the local adiabat is hotter by 100 C or more, as implied here as an alternative, what is the effect of a hotter (and presumably deeper) plume that penetrates the proposed wet MTZ? For the paper to fully address the proposed wet MTZ source, it must account for the effect of a deeper hot plume penetrating through the MTZ. If an upwelling deeper plume that penetrates and interacts with the proposed wet MTZ produces significantly different outcomes inconsistent with observations, then that would substantially support the authors' point. At the very least, this needs to be discussed in the supplement.

R: We thank the reviewer for raising this important and thought-provoking scenario. The question of how a deep, hot mantle plume might interact with a pre-existing hydrated MTZ is indeed interesting, and we agree that it deserves discussion.

If a deep thermal plume were to rise from the lower mantle and encounter a hydrated MTZ, several outcomes are plausible. 1) The excess heat could further reduce the viscosity of the already weakened, water-rich MTZ material, potentially enhancing upwelling rates. 2) Higher temperatures would also increase melt fractions, both during dehydration melting at the MTZ and during subsequent decompression in the upper mantle. 3) Geochemically, such a scenario might produce magmas that record mixing between the deep plume component (e.g., primitive He isotopes) and the hydrated, recycled component stored in the MTZ.

Whether such a hybrid scenario is required for the Azores remains an open question. The modest thermal excess ($\Delta T \leq 75°C$) inferred for Azores lavas is lower than typical estimates for deep thermal plumes. In addition, there is no clear seismic evidence for a deeply rooted mantle plume (see Fig. S1). Our primary aim is not to exclude deep plumes entirely, but to demonstrate that a deep plume is not necessary to explain the available observations. The Azores data are equally, if not more, consistent with a model in which volcanism is driven solely by hydrous upwelling from the MTZ. The hybrid plume-MTZ scenario, while plausible in principle, introduces additional complexity that is not required by the current dataset.

Following the reviewer's suggestion, we have added a discussion of this hypothetical interaction to the Supplementary Information (Text S1), in which we outline possible outcomes and acknowledge that future work would be valuable for testing such scenarios.

137-141. As written, the text takes a leap of faith. First, it mentions the evidence for a thin layer of low seismic anomalies above the MTZ. That is fine. But the next sentence, "…This hydrous melting enables widespread intraplate volcanism…", presents as evidence the thing the authors are trying to prove (a link between deep wet MTZ and shallow mantle melting). The authors have not sufficiently explained the gap between a wet MTZ top at ~ 410 km and melting at 120 km. There is nearly 300 km of mantle in between, where, somehow, the proposed hydrous melt at the top of the MTZ is creating a new source that will then melt at shallower depths. This part needs more explanation.

R: We thank the reviewer for highlighting this logical gap in the original text. The reviewer is correct that the sentence as written implied a direct connection without explaining the intermediate steps.

As we detailed in our response to your comment before, the physical process involves decompression melting of the same upwelling material, not melt migration from 410 km to 120 km. At the top of the MTZ (~410 km depth), the hydrated peridotite experiences only minor partial melting (melt fraction ≤1%). At such low fractions, the melt remains interconnected but cannot escape the solid matrix. This partially molten material is buoyant and, when entrained by ridge-induced suction, rises adiabatically. As it ascends, decompression progressively increases the melt fraction. Only when the material reaches shallower depths does the melt fraction exceed the extraction threshold, at which point the melt is segregated and emplaced at the surface to form new crust. There is no need for melt to travel independently through 300 km of mantle; instead, the solid + melt mixture rises together, and melting intensifies with decompression.

We have added a relevant description in the Methods section (Lines 500-508).

Text in 152-155 is just speculative.

R: We have removed it in the revised MS.

163. Which plateaus?

R: The original sentence was indeed problematic: it attempted to generalize our model to other plateaus without providing specific examples or acknowledging limitations.

In the revision MS, we have removed the reference to unspecified plateaus and revised the text to focus on what our model demonstrates. Specifically, our model shows that hydrous MTZ upwelling can sustain spatially and temporally distributed volcanism over ~10 Myr timescales beneath the Azores, consistent with the observed duration of platform construction (Gente et al., 2003). Whether this mechanism applies to other plateaus, such as the Galápagos (where platform volcanism lasts ~5 Myr; O'Connor et al., 2007) or Amsterdam-St Paul (~10 Myr; Maia et al., 2011), remains an open question that would require detailed geochemical and geophysical data (e.g., water content measurements, seismic imaging of the MTZ) that are currently unavailable for most

localities. We now state this more cautiously, acknowledging that our model offers a potential framework for reevaluating other plateaus, but that detailed testing awaits future data.

We have revised the text (Lines 204-209):

"Similarly, our model demonstrates that hydrous MTZ upwelling can sustain spatially and temporally distributed volcanism over ~10 Myr timescales beneath the Azores, consistent with the observed duration of platform construction (Cannat et al., 1999). This mechanism may offer a framework for reevaluating the genesis of other oceanic plateaus (such as Galápagos and Amsterdam-St Paul), particularly those formed in near-ridge settings, although detailed testing awaits comparable geochemical and geophysical datasets."

165-167. This speculative sentence basically says that everything is possible. Not very satisfying.

R: We agree that the original sentence was too vague. It attempted to generalize our results without sufficient specificity. We have deleted this sentence.

170-171: "…mantle rehydration via MTZ dehydration melting…". I think this is a key mechanism that requires further explanation.

R: We thank the reviewer for highlighting this phrase, which indeed describes a critical step in our proposed model. We apologize for not explaining it clearly.

The MTZ contains water-rich minerals (wadsleyite, ringwoodite) that can store up to 1–3 wt.% H_2O. When this material upwells across the 410-km discontinuity, it undergoes a phase transition from wadsleyite to olivine. Because olivine has a much lower water solubility than wadsleyite, this phase change triggers dehydration melting: the excess water exsolves, forming a small fraction of hydrous melt.

This low-degree melt (≤1–2%) is buoyant and water-rich. As it ascends, it interacts with and metasomatizes the overlying upper mantle, effectively rehydrating a column of mantle above the MTZ. This metasomatized mantle now has a lowered solidus and will produce significant decompression melting if entrained in further upwelling (e.g., by ridge suction). Thus, the MTZ acts as a water reservoir, and dehydration melting is the mechanism that transfers that water upward to fertilize the shallow melting region.

In the revised MS, we have added an explanation of this process in the main text (Lines 221-226).

Regarding the hydrogen isotopes. In our model, the wet heterogeneity at the top of the MTZ may represent mantle metasomatized by deep subduction-related fluids, which may be characterized by δD as low as -60 ‰ at the depth of the MTZ (Kuritani et al., 2021). However, no unambiguous sample originating from the MTZ has yet been identified for which δD has been directly measured. As this heterogeneity upwells, melting is expected to occur when water-rich wadsleyite (with high H2O solubility) transforms to olivine (with much lower H2O solubility) at the 410 km

discontinuity, and crosses the wet solidus (e.g., Bercovici and Karato, 2003; Karato, 2020; Drewitt et al., 2022). Hydrogen isotope fractionation may occur at low degrees of melting (<2%; Bindeman et al., 2012) during the formation of buoyant, water-rich melts (e.g., Drewitt et al., 2022; Xie et al., 2025). These melts may exhibit slightly higher δD values (by 10‰ to 15‰) than melts produced at degrees of melting greater than 5%, which are expected to have δD values closer to that of the metasomatized mantle source. The magnitude of this fractionation, however, remains uncertain because the $\Delta D_{melt-lherzolite}$ fractionation factor is not experimentally constrained (Bindeman et al., 2012). Hydrogen isotope fractionation may also depend on the redox state of the metasomatized heterogeneity at the top of the MTZ. Although the redox conditions of the MTZ remain poorly constrained, estimates of fO_2 range from approximately IW to IW + 3 (near FMQ; Kiseeva et al., 2018). Inclusions in some superdeep diamonds suggest that parts of the MTZ may be locally strongly reduced (e.g., Smith et al., 2016), potentially favoring the formation of molecular hydrogen (H2) (Hirschmann et al., 2012). If so, diffusive loss of H2 over time (Moine et al., 2020) could modify the local MTZ δD signature, perhaps making it less negative than that of the wet heterogeneity considered in our model. The wet molten plume with a heavy signature propagates and subsequently mixes with the overlying depleted ambient mantle (−100 ‰≤ δD ≤ −60‰; Dixon et al., 2017; Loewen et al., 2019), which could account for the range of δD values observed in Azores basalts (Dixon et al., 2017). This wet heterogeneity may represent the AP metasomatized mantle end-member proposed by Dixon et al. (2017). A further discussion is out of the scope of this study.

178: What alternative does it offer? It still requires a plume (high 3/4He or primordial He as the authors say) + recycled materials. That has always been the explanation. Please clarify.

R: The reviewer is correct that classical plume models invoke deep, primordial reservoirs (high $^3He/^4He$) plus recycled materials to explain OIB geochemistry. Our model offers a different spatial and mechanistic framework for storing and sampling these components.

In the classical view, primordial He resides in a deep, hot reservoir (e.g., the CMB) and is transported upward by a thermally buoyant plume. Recycled materials are mixed in along the way. In our model, both components can coexist within the MTZ. Super-deep diamonds from the MTZ provide direct evidence for this: they carry wide-ranging Sr-Pb-$\delta^{13}C$ isotope signatures (recycled components) coupled with extremely high and variable $^3He/^4He$ ratios (0.7–49.9 R_A). This demonstrates that a primordial, less-degassed helium reservoir has infiltrated the MTZ and resides alongside recycled material.

We propose that hydrous upwellings originating from the MTZ, driven by dehydration melting induced by ridge suction (as well as far-field subduction, e.g., Yang et al., 2025, Natl. Sci. Rev.) rather than thermal buoyancy, can sample this mixed reservoir and transport both primordial and recycled signatures to the surface. Thus, the same geochemical observations (high $^3He/^4He$ + recycled isotopes) can be explained without invoking a deep, hot plume anchored to the CMB. The alternative is not the absence of primordial material, but a different source location and mobilization mechanism.

In the revised MS, we have clarified this distinction (Lines 232-237) to explicitly state that our model offers an alternative origin and transport mechanism for the same geochemical components, rather than claiming that those components do not exist.

"In our model, hydrous upwellings originating from the MTZ, driven by dehydration melting and ridge suction, not deep thermal buoyancy, can sample this mixed reservoir and transport both signatures to the surface. This offers an alternative to classical plume models: the same geochemical observations are explained by a different source location (MTZ rather than CMB) and mobilization mechanism (hydration-driven upwelling rather than thermal buoyancy), without requiring a deep, hot plume anchored to the CMB."

We thank the reviewer for the thorough and constructive comments, which significantly help us to clarify many important issues, and the presentation of the manuscript.

Reviewer #2 (Remarks to the Author):

The manuscript entitled "Subduction legacies in the mantle transition zone modulate intraplate oceanic volcanism" by Yang and co-authors deals with the problem of the origins of oceanic plateaus – and in particular the Azores Plateau – and ocean island volcanism in what concerns their origin by mantle plumes, which the authors argue could also be explained by migration of plates over hydrous sections of the Mantle Transition Zone, which in turn they argue is the legacy of previously subducted slabs. The paper thus deals with a relevant, timely and hot topic in geodynamics, and as such brings a refreshing and different perspective to the debate. In support of their scientific discourse, the authors use model results derived from the widely used I2VIS, two-dimensional (2D) thermo-mechanical geodynamic numerical code developed by Taras Gerya and David Yuen, relating their observations with other independent observations from the literature, namely on the water content and geochemical signature of melts. Personally, I enjoyed reading the manuscript and was encouraged by the novel perspective. If the authors are right, what they propose is far-reaching and with key consequences for what we know about the formation of oceanic plateaus, island volcanism and mantle melting. I think however, this study is not yet at the state of maturity to offer a solid contribution to the debate despite its excellent potential, particularly taking into account the high standards of Nature Communications. I therefore recommend major revisions and leave here a few aspects as food for thoughts in what I consider to be weaknesses or points for improvement in the paper.

R: We thank the reviewer for his thorough and constructive assessment of our manuscript. We are encouraged by the reviewer's positive remarks. We also appreciate the reviewer's honest evaluation that the manuscript in its current form is not yet mature enough for publication in Nature Communications. We fully agree with this assessment and are committed to addressing the specific weaknesses and points for improvement identified in the subsequent comments. Please see our revised MS and response to your comments below.

[1] first and foremost, I find the way the model results are displayed in figure 2 – which is absolutely central to the paper – really difficult to read, making it hard to see how what is observed in the figure may or may not support the far-reaching statements found in the text. This is partially due to the choice of colours, lack of velocity field vectors in the entirety of the model panels (limiting the reader's capacity to understand and interpret the data using the main figures of the paper), difficulty in reading the isothermal lines and labels, and also the differentiation of the different fields displayed in the figure, which I find really confusing (see my comments on the manuscript). For the manuscript to be published in Nature Communications I suggest the authors overall the way they display the model and significantly improve the readability of the figure, which should be self-explanatory and at this stage (perhaps is just me) requires quite a lot time to digest and understand.

R: We thank the reviewer for this constructive and detailed feedback on Fig. 2. We agree completely that this figure is central to the paper and must be clear and self-explanatory. In response, we have redrafted Fig. 2, and major changes include:

1) We have now added velocity vectors across all model panels.

2) We have improved the color scheme. The hydrated mantle is now shown a distinct color, while we removed the isotherms (moved to Fig. 3) and the extracted melt fraction (moved to Fig. S4) in this figure to facilitate clarity.

3) We have added an additional figure for the melt fraction and overlaid with isotherms.

4) We have added a new supplementary figure (Fig. S4) showing the evolution of water content, viscosity, and mantle depletion through time. This provides readers with additional context to understand the dynamics depicted in Fig. 2.

[2] second it is not clear to the reader – just on the basis of this figure – how exactly the model supports the formation of a plateau such as the Azores plateau - you need to find a more effective way to link the model runs (perhaps through a more effective labelling) to make this link more evident for the less specialist readership. In this respect the video seems to be a bit more evident. I acknowledge that I am not geodynamic modeller but more so the model should be better comprehensible (or more readable) by a less niche reader such as me.

R: We thank the reviewer for this important feedback. We understand that Fig. 2 must stand alone and be interpretable by readers who are not geodynamic modeling specialists. We have the following modifications to make the connection of this figure to the Azores Plateau:

1) in the zoomed insets, we explicitly label the thickness (~12 km) of newly formed oceanic crust (Fig. 2e), and distinguish the origin of the newly formed crust (from either dry mantle melting or wet mantle melting in Fig. 2f).

2) We have added text labels for the key stages: 1. initial partially molten wet mantle -> 2. hydrous upwelling -> 3. ridge-induced suction and enhanced melting -> 4. thick crust formation.

3) The labeled time elapsed (from 35.8 to 53.8 Myr is somewhat similar to the duration of the plateau formation in the Azores.

[3] third, if I understood the model correctly, the model implies hydrous melting and hydrous volcanism at the MOR but only for a period (presumably when the plateau is generated) – where is the evidence for that? I.e. where is the evidence that the lavas that formed the Azores plateau are particularly rich in volatiles and as such reflect an hydrous mantle source? And notwithstanding the fact that many Azores Island lavas (but not the plateau lavas, from my knowledge) reflect a volatile-rich source, there is also evidence from noble gas geochemistry suggesting a thermal (or thermochemical) mantle plume (e.g. Madureira et al, 2005; Madureira et al., 2014; Moreira et al 2018)... how do you conciliate these different pieces of evidence?

R: We thank the reviewer for raising these two important points.

1) As for the 1st comment on the evidence for volatiles in plateau lavas. Direct H_2O measurements from the plateau lavas are indeed limited, but critical evidence comes from Métrich et al. (2014), who analyzed melt inclusions from Pico Island (the youngest island) and found high H_2O contents (up to 2.0 wt%) and elevated H_2O/Ce ratios (259 ± 21). Crucially, they note that these values are similar to published ratios for submarine basalts from the Azores platform. Thus, the available data (though sparse) indicate that the plateau source was similarly hydrated. This is consistent with Asimow et al. (2004), who modeled that much of the crustal thickness anomaly at the Azores platform can be explained by a hydrous source (up to ~700 ppm H_2O) with only a modest thermal anomaly ($\Delta T \leq 75°C$). Beier et al. (2012) further show that Azores lavas require at least 200 ppm H_2O in the source because their melting temperatures are lower than the dry mantle solidus. We have added a brief discussion in the revised MS (Lines 142-151).

"Crucially, evidence for a volatile-rich mantle source extends to the plateau lavas themselves. Métrich et al.6 analyzed melt inclusions from Pico Island and found elevated H_2O content (up to 2.0 wt.%) and H_2O/Ce ratios (259 ± 21). Most importantly, they note that these values are similar to published ratios for submarine basalts from the Azores platform (253 ± 33)35, indicating that the plateau source was similarly hydrated. This is consistent with Asimow et al.36, who modeled that much of the crustal thickness anomaly at the Azores platform can be explained by a hydrous source (up to ~700 ppm H_2O in the mantle) with only a modest thermal anomaly ($\Delta T \leq 75°C$). Beier et al.7 further show that Azores lavas require at least 200 ppm H_2O in the source because their melting temperatures are lower than the dry mantle solidus."

We further have additional text in the Supplementary Material (Text S2) to describe the model limitations.

2) As for the 2nd comment on the noble gas evidence. The relatively high $^3He/^4He$ ratios (up to ~14.8 RA) in some Azores lavas have been interpreted as evidence for a lower mantle plume (Madureira et al., 2005, 2014; Moreira et al., 2018). Our model offers an alternative explanation that does not require a deep thermal plume. Super-deep diamonds from the mantle transition zone preserve both recycled isotopic signatures and primitive $^3He/^4He$ ratios (0.7–49.9 R_A; Timmerman et al., 2019), demonstrating that primordial helium resides in the MTZ alongside recycled material. In our model, hydrous upwellings originating from the MTZ can sample this mixed reservoir and transport both signatures to the surface. Thus, the noble gas data are not incompatible with our mechanism, and they simply relocate the primordial reservoir from the lower mantle to the MTZ. We have added a brief discussion of this in the revised MS (Lines 232-237)

[4] more broadly – and more importantly due to the implications the arguments bring - your model basically implies that most intra-plate volcanism can me potentially explained by melting taking place in hydrous zones of the MTZ... at least this is what the title and some statements imply. How do you conciliate this with evidence from melt geochemistry and seismic tomography at other intra-plate localities that strongly suggest the presence of mantle plumes deeply anchored in the CMB and for melting to reflect primitive rather than recycled sources (e.g Iceland, Hawaii, Canaries/Cape Verde)? I particularly feel this part of the debate in the paper is not fully supported by evidence from the literature..

R: We apologize if our wording gave the impression that we are proposing this mechanism as a universal replacement for mantle plumes. That was never our intention, and we have revised the manuscript to avoid any such overreach.

We do not dispute that many well-studied intraplate localities (e.g., Hawaii, Iceland) show compelling evidence for deep-rooted thermal plumes, including excess mantle temperatures, age-progressive volcanism, and seismic anomalies extending to the lower mantle. Our model is not intended to challenge these cases. Instead, considering some regions lacking evidence for mantle plumes anchored at the CMB (for example, the seismic anomalies in Fig. S1), our aim is to demonstrate that in specific settings where a migrating ridge interacts with a localized, hydrated domain in the MTZ, intraplate volcanism can be generated without requiring a deep thermal plume. This mechanism has been overlooked in the literature, despite its consistency with both numerical models and growing seismological evidence of heterogeneous hydration atop the 410-km discontinuity.

We have now carefully revised the manuscript to make clear that our model offers a complementary, not competing, mechanism. The title and key statements have been softened to reflect that we are proposing an alternative explanation for cases like the Azores, not a new paradigm for ALL intraplate volcanism. The revised texts are in Lines 210-218.

[5] what the authors describe as "upwelling" from the MTZ are effectively – or geometrically – what others describe as plumes, even if these are only anchored at the MTZ rather than the CMB... Therefore I think the use of the terms here is perhaps not entirely appropriate although I partially understand why the authors avoid using the term "plumes" to describe the (presumably columnar) upwelling they observe in the their models.

R: We agree that the upwellings in our models are geometrically consistent with the common understanding of mantle plumes. As correctly noted by the reviewer, our reluctance to use the term "plume" stemmed from a desire to avoid confusion with the classical, deep-rooted thermal plumes anchored at the CMB. We have explicitly defined the classical thermal mantle plume in Lines 31-33.

We now use the term "hydrous upwelling" throughout the text to describe the features in our models.

I leave these comments here as some food for thought, hopefully they all will help improve a manuscript that despite being (in my view) not yet ready for publication, when it is ready and improved may stir the debate on the formation of oceanic plateaus and ocean island volcanism, providing a provocative alternative explanation to these features.

R: We sincerely thank the reviewer once again for your thoughtful and constructive comments, which have helped us substantially improve the manuscript. We appreciate the encouraging words and the open-minded recognition that our work may "stir the debate" on the formation of oceanic plateaus. We have carefully addressed all the points raised and believe the revised manuscript is now much stronger and clearer. We hope it meets the reviewer's expectations and the high standards of Nature Communications.

[Remarks from the annotated PDF]

Line 38: In this case, I don't see how the lack of age-progressive volcanism challenges the presence of a hot plume, given that the emplacement of the emplacement of the palteau does not need to be -age-progressive and most island volcanism in the Azores is, to some extent, tectonically controlled, with volcanism occurring mostly along fissures and clear tectonic structures such as the Terceira Rift (and therefore without a need for age-progression)...

R: We thank the reviewer for the comment. We did not intend to suggest that plateau emplacement itself should be age-progressive. The classical "stationary plume hypothesis" predicts a clear age progression in hotspot tracks (the chain of islands and seamounts formed as the plate moves over a fixed plume), not necessarily in the initial plateau-forming phase. Our original wording was imprecise and could be misread.

Now we have revised the sentence to clarify that the lack of a clear, long-term age-progressive track and seismic anomalies down to the CMB, combined with overlapping eruption ages across the archipelago, is what challenges the classical plume prediction, while acknowledging that plateau emplacement and tectonic controls can complicate the expected pattern. See Lines 39-42.

Lines 40-41: Indeed, but this evidence was mostly derived from island lavas not plateau lavas... what evidence do you have that the plateau was formed by volatile-rich melts? Also, importantly, noble gas geochemistry (He and Ne isotopes) of the Azores Islands lavas, albeit with a lot of variability, is more compatible with the presence of a thermochemical plume beneath the Azores... how do you conciliate these 2 pieces of evidence? Could you elaborate more on this?

R: Please see our detailed reply to your Comments #3 and revised text (Lines 39-42, and Lines 123-130).

Lines 44-45: I agree!

R: Thanks.

Lines 75-76: why does the Rayleigh-Taylor instability only develops at this stage? Could you add more detail here or perhaps vectors in figure 2a-d that illustrate mantle flow outside the domains where these are already present?

R: The Rayleigh-Taylor instability develops only when the hydrated layer has been sufficiently entrained and thinned by ridge-induced corner flow to become gravitationally unstable. The exact timing depends on model parameters such as the water content, layer thickness, and layer width (Fig. S6). For our reference model (0.3 wt.% H_2O, 75 km thick), the instability becomes visible at ~35.8 Myr, when the ridge has migrated close enough to the hydrated domain to generate sufficient suction. We have added the velocity vectors across panels b–d, as well as a description of the caption in Fig. 3.

Lines 78-81: Again, the figure would benefit from having more vectors to illustrate mantle flow beyond the domains already represented... More importantly, according to the figure, we should expect hydrous melting at the MOR around 53.8 Ma, if I understood the figure correctly – where is the evidence for that and what would be the expected consequences of such hydrous melting?

R: We have added the velocity vectors. We suspect there is a misunderstanding about the timing. The value “53.8” in Fig. 2b refers to model time in Myr after the start of the simulation, not to geological age (Ma). This is the time elapsed since the model run.

At 53.8 Myr model time, our simulation predicts hydrous melting beneath the ridge axis, manifested as a secondary finger-like upwelling (Fig. 2c). The expected consequence is the production of thicker-than-normal oceanic crust (~12 km in our model) with elevated water contents in the erupted basalts. This prediction is consistent with observations from the Azores platform, where: (1) seismic studies reveal crustal thickness of 8–30 km; (2) MORB from the Azores platform exhibit elevated H_2O/Ce ratios compared to background Atlantic MORB (Fig. 1b); and (3) petrological modeling indicates a hydrated mantle source (Asimow et al., 2004; Metrich et al., 2014). Thus, the model predictions align with the available observations from the Azores.

Lines 81-83: Again, using the Azores case-study, where is the evidence for this?

R: We must be honest that there is currently no direct observational evidence from the Azores that confirms this specific model prediction. The asymmetry between hydrous melting beneath the trailing flank and dry melting beneath the leading flank is a prediction of our geodynamic model, but testing it would require recovering and analyzing basalts from both flanks of the paleo-ridge that formed during plateau construction. However, the samples are not currently available.

We further clarify that this is a model prediction awaiting confirmation (Lines 92-93):

“…This asymmetry is a prediction of our model that awaits testing with future geochemical sampling of the plateau's conjugate flanks…”

Lines 104-106: and where is the petrological/geochemical evidence for that?

R: We kindly ask the reviewer to refer to our reply to Reviewer #1's comments #1, #3, #4, and the revised text in the section "Implications for the thick crust in the Azores Plateau" (Lines 113-130)

Lines 116-117: a citation should support this statement, if possible

R: We have added Smyth & Jacobsen (2006) and later Litasov & Ohtani (2007), Frost (2008).

Lines 139-141: does this mean that all site of intra-plate volcanism can be explained by hydrous melting derived from the MTZ? and why would we expect these sites of upwelling to be stationary over time? Anyway, the model still requires upwelling (i.e. plumes) of chemically and possibly thermally buoyant material which is in effect a mantle plume, just not anchored at the CMB but at the MTZ... the wider implications of what is being proposed (i.e. beyond the Azores case study(are profound and should be discussed better/further...

R: We kindly ask the reviewer to see our response to your Comment #4.

Figure 1a: First, none of the squares is red (1 is orange and 3 are yellow); second, none of these locations is above particularly wet MTZ – so how to explain the signature at the surface?

R: Thanks for pointing out our mistake regarding the color issue, which has been corrected in the caption.

Regarding the second question: we use Fig. 1a to illustrate that present-day MTZ water content is heterogeneous, with some regions showing elevated values. However, we do not suggest that present-day hydration directly correlates with past volcanism at these exact locations. Instead, we highlight that those three locations (Bermuda, Equatorial Atlantic, South China Sea) have been independently linked to water-rich volcanism and/or MTZ dynamics. For example, 1) Bermuda: Mazza et al. (2019) proposed a volatile-rich MTZ source for Bermuda volcanism based on geochemistry. 2) Equatorial Atlantic: Ligi et al. (2005) found water-rich basalts at the ridge, and Agius et al. (2021) imaged a thinned MTZ beneath this region, interpreted as ridge-induced upwelling (this process is remarkably similar to our model). 3) South China Sea: Qian et al. (2025) linked MTZ water to lithospheric weakening and spreading in this region.

These observations are consistent with our proposed mechanism: ridge suction can mobilize hydrated MTZ material, generating upwellings that produce water-rich magmatism at the surface. The Azores, though not highlighted in Fig. 1a due to limited MTZ water estimates, shares the same characteristics (water-rich basalts and proximity to a migrating ridge), which makes it an ideal test case. We have added a brief discussion of these independent examples in the revised manuscript (Lines 181-184) to strengthen the broader applicability of our model.

Figure 1b: what about oceanic plateau lavas? could you also plot OIB and plateau lavas for comparison, next to this plot?

R: We thank the reviewer for this suggestion. Unfortunately, direct H_2O measurements for Azores Plateau lavas (the off-axis platform basalts) are not available in the published literature. For OIB from the islands, the only published water data we are aware of are from Pico Island (Métrich et

al., 2014), which yield H_2O/Ce ratios of 259 ± 21—within the range of the on-axis MORB shown in Fig. 1b. Métrich et al. (2014) further note that these values are "similar to published ratios for submarine basalts from the Azores platform," suggesting that the plateau source was similarly volatile-rich, even though direct measurements are lacking. We now mention this in the main text (Lines 142-146) that available OIB data from Pico Island are consistent with the elevated H_2O/Ce ratios observed in Azores MORB, supporting a consistently hydrated mantle source across the region.

"Crucially, evidence for a volatile-rich mantle source extends to the plateau lavas themselves. Métrich et al.[6] *analyzed melt inclusions from Pico Island and found elevated H_2O content (up to 2.0 wt.%) and H_2O/Ce ratios (259 ± 21). Most importantly, they note that these values are similar to published ratios for submarine basalts from the Azores platform (253 ± 33)*[35]*, indicating that the plateau source was similarly hydrated."*

Figure 2: I confess I find this figure – which is absolutely central do the argumentation in the paper – extremely difficult to read and understand... this is mostly due to a poor choice of colors and the absence of mantle flow velocity vectors away from the domains represented and partially due to the scale of the figure. Critically the figure also is confusing because it mixes melt presence, melt extraction, wet/dry mantle... also, what do you mean by "wet or dry molten mantle? the mantle is never fully molten...do you mean melt-rich mantle? could you please clarify?

R: We kindly ask the reviewer to see our response to your comment #2 above.

Regarding the terminology, the reviewer is correct that "molten mantle" was imprecise. We have revised this to "partially molten dry/wet mantle" throughout the figure and caption. "Dry mantle melting" refers to partial melting of ambient, water-poor mantle, while "wet mantle melting" refers to partial melting of the hydrated domain. This has been corrected in the figure legend.

Figure 2b: what is happening here – could you please represent the velocity field across all figure? the same for other panels...

R: Now we have added velocity vectors for panels b–d.

this upwelling is, in all effects, a...erg... a plume, just not anchored at the CMB but at the MTZ.

R: We agree with the reviewer. The upwellings in our models are geometrically and dynamically akin to plumes, but they originate from the MTZ rather than the CMB and are driven by hydration rather than thermal buoyancy. To avoid confusion with classical thermal plumes, we now use the term "hydrous upwelling" throughout the manuscript to describe these features.

I guess this sharp boundary between wet mantle and wet molten mantle is imposed by an isotherm – could you please represent it?

R: We thank the reviewer for this observation. The sharp boundary in the original figure reflected the extracted melt fraction from the hydrated domain, not an isotherm. In the revised Fig. 2, we have improved the visualization by 1) using a more intuitive color for the wet mantle, 2) removing

the color-scaled field of extracted melt fraction (moved to Fig. S4) and adding specific labels (see also Fig. S4 for residual water contents). The isotherms are now moved to the new figure (Fig. 3 in the revised MS).

Figure 2c: This implies hydrous melting and hydrous volcanism at the MOR for a sustained period of time... where is the evidence for that? to same extent the same applies in panel d.... where is the evidence for hydrous melting and volcanism in the Azores plateau? Also, can you make a clear reference in the model of the timing of formation of the plateau and its cessation afterwards? This is not clear to me...

R: In Fig. 2c and 2d, the mantle beneath the ridge experienced melt extraction, leaving it with very low residual water contents (<0.00001 wt.% H_2O; see Fig. S4l). We now label this region as "very low water content after melt extraction (Fig. S4l)" in the revised Figure 2 to clarify that it is effectively dry.

Regarding evidence for sustained hydrous melting: direct H_2O measurements from off-axis plateau lavas are not available. However, Pico Island OIB (Métrich et al., 2014) yields H_2O/Ce ratios (259 ± 21) within the range of on-axis Azores MORB (Fig. 1b), suggesting that the mantle source is hydrated where volcanism is currently active. The sustained hydrous melting in our model is a prediction that awaits testing with future geochemical sampling of the plateau's conjugate flanks.

Yours truly,

Ricardo Ramalho

Reviewer #3 (Remarks to the Author):

In this work, Yang and co-workers challenge the classic mantle plume origin for oceanic plateaus by proposing an alternative mechanism, namely that a hydrated mantle transition zone (MTZ) can create localized upwellings of increased melt, which leads to long-term volcanism and anomalous crustal thickness.

Their results seem to validate this mechanism, where the formation of localized upwelling derived due to adiabatic decompression in the vicinity of the mid-ocean ridge can be seen. According to the authors, these results are similar to what can be observed in the Azores Plateau.

The manuscript was quite interesting to read, but I do have some questions and comments regarding their work:

1. While I agree with the authors' assessment that the classic mantle plume model (i.e., a deep-rooted thermal anomaly, stemming from the core-mantle boundary) does not explain the observed anomalies at the Azores, I do believe that what they model and show is, for all effects and purposes,

a plume. It is described by the authors as a Rayleigh-Taylor instability which upwells to the surface (ln. 75-77), which is exactly what a thermal plume would do. In fact, this is clear by observing Fig. 2, where two clear mushroom-like anomalies ascend through the upper mantle. In my view, this work contributes towards expanding our understanding of mantle plumes, showing a different way of forming them at lower depths along the MTZ and I believe the authors should lean into this aspect. If they disagree, I would at least like to know their opinion.

R: We thank the reviewer for the encouragement and thoughtful observation.

As our reply to Reviewer #2, we agree that the upwellings in our models are geometrically and dynamically akin to plumes, but they originate from the MTZ rather than the CMB and are driven by hydration rather than thermal buoyancy. To avoid confusion with classical thermal plumes, we now use the term "hydrous upwelling" throughout the manuscript to describe these features. We have added a relevant description in Lines 31-33.

2. On the topic of looking at figures, I don't entirely follow the authors' reasoning with Figure 1. The authors state that there are "elevated H2O/Ce ratios of both OIBs and MORBs from the Azores compared to background MORB ratios to the south" (ln. 42-43), however there doesn't seem to be a significant difference between the values for the Azores and the rest of the data points. In fact, they seem to plot very much along the trend shown. Given that the increased water content seems to induce upwellings, following this reasoning I would expect significant intraplate volcanic activity in the mid Pacific. Could the authors comment on this? Also on this figure, there are no red squares as indicated by the caption, probably a typo.

R: We thank the reviewer for pointing out our color typo. The reviewer is correct that the caption incorrectly referred to "red squares." We have corrected this to " yellow squares" and "pink square" to match Fig. 1a.

We have redrafted Figure 1b to highlight the Azores peak more. The water contents show a more pronounced peak in the Azores (Fig. S2). The H_2O/Ce ratios may be a more robust proxy for the mantle source water than H_2O.

Critically, multiple independent studies confirm that Azores magmas are indeed water-rich. Asimow et al. (2004) modeled Azores MORB and concluded that they require a hydrous mantle source with up to ~700 ppm H_2O. Métrich et al. (2014) analyzed melt inclusions from Pico Island and found elevated H_2O contents (up to 2.0 wt%) and H_2O/Ce ratios (259 ± 21), noting that these values match those of the Azores platform basalts. Please also refer to the reply to Reviewer #2 (Comment 3).

The reviewer raises an important question: if elevated H_2O/Ce alone induced upwellings, why don't other high-H_2O/Ce locations along the ridge (e.g., the equatorial Atlantic; Ligi et al., 2005. We think you're referring to mid-Atlantic, not mid-Pacific) host comparable volcanic plateaus? We suggest that there are specific requirements for a thick plateau: 1) Water content alone is not sufficient. The degree of hydration must be high enough to generate sufficient buoyancy and melt productivity (for example, 0.1 wt.% water cannot produce very thick crust, Fig. 4 & Fig. S6a).

Whether the equatorial Atlantic hydrated domain reaches the threshold required for plateau formation (e.g., ≥0.1 wt% H_2O in the source over a sufficiently large area) remains unknown.

2) The other reason is that a migrating ridge must pass over the hydrated domain to entrain and mobilize it. We have added a relevant description in the section "Implications for the thick crust in the Azores Plateau" (Lines 160-165).

"The scarcity of thick, plateau-like crust along most mid-ocean ridges may reflect the heterogeneous distribution of water in the MTZ. Only where a sufficiently large and highly hydrated domain interacts with a migrating ridge, as proposed for the Azores, does this process generate enough melt to produce a plateau-scale edifice. Smaller or less hydrated domains may produce more subtle anomalies, while larger domains could, under favorable tectonic conditions, generate even more massive features."

3. Continuing to Figure 2, I find the colour legends applied to be unclear. What applies to what? It appears to be a superposition between the composition colours and the melt fractions which makes it unclear to me. This makes it quite hard to observe the crustal thicknesses described in the text. This could be solved by adding a scale to the insets on the right.

R: Indeed, as pointed out by other reviewers, Fig. 2 is overly complex and unclear. We now make these changes:

1) We show the melt fraction field in a new figure (Fig. 3), and the extracted melt fraction is shown in the supplementary figure (Fig. S4).

2) We have added explicit labels in Fig. 2 to identify key features, including "hydrous upwelling," "depleted wet mantle," and so on.

3) In the zoomed insets (now labeled as panels 2e and 2f), we have added a label to indicate the crustal thickness (~12 km), as well as the length scale for "10 km", and indicated the origin of the crust (either from wet or dry mantle extraction).

4. Still in Figure 2, on 2c) and onwards, I see that there is a region of zero melt wet mantle within the plumes, between ca. 100 and 180 km deep. What is the reason for this? The linear aspect of it suggests that it might be a phase transition or pressure effect, but I couldn't find an explanation for it in the manuscript.

R: We thank the reviewer for this careful observation. The feature they identify (the greyish-white region between ~100–180 km depth) represents depleted mantle that has already undergone melt extraction. In our model, as the hydrous upwelling rises and melts, water is partitioned into the melt and removed upon extraction, leaving behind a residuum with very low water content (<0.00001 wt.% H_2O; see Fig. S2). This depleted mantle is no longer fertile and does not produce additional melt upon further decompression, creating the "zero melt" zone that the reviewer observes.

The linear appearance is not a phase transition or pressure effect, but simply the boundary between previously unmelted (fertile) and melted (depleted) mantle. To clarify this in the revised figure:

We have added the label "depleted mantle after melt extracted" (to indicate the depleted mantle, rather than using the color scale for its depletion, as shown in the supplementary Fig. S4) directly to Fig. 2c and 2d to identify this region. We have added a sequential guide to the figure caption that walks readers through the process:

1. initial partially molten wet mantle
2. hydrous upwelling
3. ridge-induced suction and enhanced melting
4. thick crust formation

5. Later stages of the authors' model show an upwelling within the mid-ocean ridge (MOR) which, in my view, would have to imply result in water-rich basalts being formed at the MOR itself. I saw some references in the ms to water-rich basalts in cold spots of the Mid-Atlantic Ridge (ref. 40 I believe?), but this is significantly further south. Is there evidence for hydrated basalts at the MOR in the Azores? If not, the authors should discuss this somewhere, perhaps in a model limitations section.

R: Azores MORB exhibit elevated H_2O/Ce ratios compared to background Atlantic MORB south of the region (Dixon et al., 2017; Metrich et al., 2014; Zhou et al., 2023). These data come from on-axis basalts recovered from the Mid-Atlantic Ridge segment adjacent to the Azores Plateau. Asimow et al. (2004) further demonstrated that these MORBs require a hydrous mantle source with up to ~700 ppm H_2O to explain their composition and the observed crustal thickness, with only a modest thermal anomaly ($\Delta T \leq 75°C$).

Following the reviewer's suggestion, we have added a brief discussion of this limitation in the Supplementary Material "Text S2 Model limitations and future directions."

6. While the authors show the thermal fields for the last stages of the model in Fig. S3, I'd like to see it for the remaining stages, particularly for 53 Myr stage. By the final stage there is a near total recuperation of the initial temperature profile (plus or minus a few tens of degree) but, from what I can see in Fig. 2c-d, the 900C isotherm here is much lower than on the other figures so I'd like to understand this discrepancy.

R: That's a great spot, and thanks for this exceptionally careful observation! We must apologize, and the reviewer is absolutely correct. There is an error in Fig. 2c–d as originally presented. The lower 900ºC isotherm in these panels should be 1400ºC. This mistake occurred during figure preparation and unfortunately slipped through our checks. We have now corrected it in the revised MS. We have also uploaded the data for the reference model (shown in Fig. 2) and GMT scripts. The Supplementary Movies and Supplementary Figures show the correct isotherms.

We are grateful to the reviewer for catching this mistake.

I believe this paper to be of great importance to the field of geodynamics; however, the additional clarifications suggested will increase its impact and clarity for the potential reader. I am confident that addressing these issues will make manuscript will be a worthy contribution to this journal.

The authors should feel free to contact me if they have any questions regarding my review.

Best regards,

Jaime Almeida

R: We thank the reviewer for the thorough and constructive review, as well as his encouraging words. The insightful comments and suggestions have helped us substantially improve the manuscript.

REVIEWERS' COMMENTS

Reviewer #1 (Remarks to the Author):

I commend the authors for their diligent work in the revision. I find that the paper now is more "honest", meaning that it explores more caveats and addresses some inconsistencies or things that were unclear. The paper provides evidence for an additional mechanism to understand intraoceanic excess volcanism, particularly one that is assisted by a migrating ridge.

The one thing that is quite pertinent in the Azores plateau, and my apologies for not thinking about in the first review, is the triple junction of the Atlantic ridge and the Terceira Rift. Work by Georgen (e.g. Georgen EPSL 2008, and some other work), have shown that the triple junction may result in excess melting as a result of the three plate spreading, and therefore excess crust production. Understandably, a 3D flow model may be significantly different than the model in this paper. Nonetheless, as the manuscript describes the excess magma at the Azores triple junction, it should also discuss that other models have attributed excess crustal production to the Triple junction configuration, albeit assisted by a nearby "hot plume". Can the authors speculate how a triple junction mantle flow affect their model calculations and if some of the excess "crust" is due to the mantle flow assisted by the Triple junction, what / how could it affect their model input (perhaps the amount of water in the source, if water drives degree of melting)?

We thank the reviewer for reminding the important role of the Azores triple junction. In this study, we propose that the low-pressure region of the mid-ocean ridge can generate a suction force that drives hydrous MTZ upwelling and mantle melting. We agree that the triple junction, as previous numerical models have shown, likely enhances our proposed mechanism. According to previous models (Georgen, 2008, 2010), the 3D divergence of three plates creates stronger mechanical suction, making it easier to tap the hydrated MTZ. We speculate that the triple junction can focus hydrous material laterally from a broader MTZ volume into the ridge system. Therefore, this focusing may reduce the required water content in our models, although the 3D focusing effect alone may not be sufficient for melting when accounting for adiabatic cooling. Our 2D models cannot capture 3D effects, but the 3D effects of triple junctions alone may not always produce a plateau (see discussion in Sager et al., 2016), and thermal/chemical anomalies are often required. Future 3D modeling that incorporates a hydrous MTZ and full triple-junction kinematics will be required to investigate the influence of source fertility and plate geometry on the formation of the Azores Plateau (as well as other triple junctions & oceanic plateaus). We have added a brief discussion in the main text (Lines 167–175).

We thank the reviewer again for the constructive and helpful comments.

Reviewer #2 (Remarks to the Author):

I am happy with the authors' response and changes in the manuscript – I believe they address all reviewers' concerns in a satisfactory manner, despite some still existing weaknesses (lack of evidence for wider spread hydrated melts in the plateau and islands – so far evidence is restricted to OIB lavas from Pico and offshore. The manuscript is solid enough to stir the debate and provide an alternative model for the formation of the Azores and the most excessive comments were toned down or modified to make them clearer. I am therefore happy for the manuscript to be published as it is.

We thank the reviewer again for the constructive and helpful comments.

Reviewer #3 (Remarks to the Author):

The authors have adequately addressed all my comments and questions. The clarity of both the text and figures of the manuscript have significantly increased during the review.

It is my opinion that the manuscript is now ready for publication.

Best regards,

Jaime Almeida

We thank the reviewer again for the constructive and helpful comments.
